# A novel designed membrane-active peptide for the control of foodborne *Salmonella enterica* serovar Typhimurium

**DOI:** 10.1038/s41598-023-30427-z

**Published:** 2023-03-02

**Authors:** Siriwan Sengkhui, Natthaporn Klubthawee, Ratchaneewan Aunpad

**Affiliations:** 1 Graduate Program in Biomedical Sciences, Faculty of Allied Health Sciences, Thammasat University, Pathum Thani, Thailand; 2 Department of Medical Technology, Faculty of Allied Health Sciences, Pathumthani University, Pathum Thani, Thailand

**Keywords:** Microbiology, Applied microbiology

## Abstract

The main cause of non-typhoidal *Salmonella* (NTS) infection in humans is ingestion of contaminated animal-derived foods such as eggs, poultry and dairy products. These infections highlight the need to develop new preservatives to increase food safety. Antimicrobial peptides (AMPs) have the potential to be further developed as food preservative agents and join nisin, the only AMP currently approved, for use as a preservative in food. Acidocin J1132β, a bacteriocin produced by probiotic *Lactobacillus acidophilus*, displays no toxicity to humans, however it exhibits only low and narrow-spectrum antimicrobial activity. Accordingly, four peptide derivatives (A5, A6, A9, and A11) were modified from acidocin J1132β by truncation and amino acid substitution. Among them, A11 showed the most antimicrobial activity, especially against *S*. Typhimurium, as well as a favorable safety profile. It tended to form an α-helix structure upon encountering negatively charged-mimicking environments. A11 caused transient membrane permeabilization and killed bacterial cells through membrane depolarization and/or intracellular interactions with bacterial DNA. A11 maintained most of its inhibitory effects when heated, even when exposed to temperatures up to 100 °C. Notably, it inhibited drug-resistant *S*. Typhimurium and its monophasic variant strains. Furthermore, the combination of A11 and nisin was synergistic against drug-resistant strains in vitro. Taken together, this study indicated that a novel antimicrobial peptide derivative (A11), modified from acidocin J1132β, has the potential to be a bio-preservative to control *S*. Typhimurium contamination in the food industry.

## Introduction

Non-typhoidal *Salmonella* (NTS) infection is a major global health problem. Each year, an estimated 93.8 million cases of gastroenteritis caused by NTS infection result in approximately 155,000 deaths^1^. NTS infection primarily results from consumption of undercooked or contaminated food or food products (primarily poultry, eggs, beef, or milk)^2^. A variety of food preservatives, particularly chemicals (such as nitrites, nitrates, and sulfur dioxide), are employed in the food industry. However, their excess use may cause adverse side effects in consumers and compromise nutritional levels of foods^3^.

Antimicrobial peptides (AMPs) are promising as alternative food preservatives due to their enhanced antimicrobial activity and safety, while maintaining nutritional and sensory qualities^3,4^. Bacteriocins are ribosomally-synthesized antimicrobial peptides or proteins produced by different genera of both Gram-positive and -negative bacteria^5^. Among them, bacteriocins from lactic acid bacteria (LAB) have attracted increasing recognition for development as food preservatives. This is because of their broad killing spectrum, synergism with other antimicrobials, high stability under food processing conditions (heat and pH), and low likelihood of cross resistance with antimicrobial agents^6^. Furthermore, a long record of safe use in foods of bacteriocins from LAB and their GRAS (Generally Recognized as Safe) status has paved the way for their development as potentially safe antimicrobials to control food spoilage caused by bacteria^7^.

Nisin, the most extensively utilized bacteriocin, is a member of the natural AMPs known as lantibiotics produced by *Lactococcus lactis* subsp. lactis^8^. It has been approved for use in food and obtained GRAS status from the U.S. Food and Drug Administration^9^. However, limiting the usefulness of nisin’s food application is its poor activity toward Gram-negative bacteria, molds and yeasts, together with loss of activity during processing and storage^10,11^. Development of a new agent against foodborne Gram negatives, especially non-typhoidal *Salmonella*, is needed but challenging. The combination of nisin with a new compound could further inhibit undesirable foodborne pathogens^12^. The combination of buforin I, a cationic AMP derived from the stomach of an Asian toad, and nisin displayed such synergism against a range of food spoilage microorganisms, including *Listeria innocua*, *E. coli*, and *Rhodotorula glutinis*^13^. A synergistic result of nisin with carvacrol was observed in pre-sliced bologna sausages contaminated with *L. monocytogenes* during 7 days of storage^14^.

More effective AMPs can be designed from originally natural AMPs by several modification strategies, notably amino acid substitution and truncation to adjust characteristics such as net charge, hydrophobicity, length, secondary structure and amphipathicity. This may lead to novel peptides with desirable properties, such as potent and broad antimicrobial activity, reduced toxicity, and improved stability^15^. There have been several successes in developing such novel AMPs through amino acid substitution and truncation^16–18^. Zhang et al.^18^ reported that the substitution of alanine (position 1), alanine (position 8) and isoleucine (position 17) with positively charged residues (arginine or lysine) retained both the antibiofilm and antimicrobial activities of the parental peptide, and considerably reduced hemolytic and cytotoxic effects toward mammalian cells.

Acidocin J1132β, a bacteriocin produced by probiotic *Lactobacillus acidophilus* JCM 1132, displays low and narrow-spectrum antibacterial activity which is most effective against closely related *Lactobacillus* spp.^5^. However, it demonstrated no or low toxicity in vitro as its producers are nonpathogenic and members of normal intestinal microflora, and was subsequently promoted as a preservative in various food products^19,20^. To further exploit it as a food preservative, modification of this peptide was investigated. In the present study, a series of novel peptide derivatives modified from acidocin J1132β by truncation and amino acid substitution were designed and synthesized, and a peptide with enhanced activity and low toxicity was selected for determination of secondary structure, cytotoxicity and mechanism of action against *S*. Typhimurium. The stability of this candidate peptide was investigated and possible synergism with nisin was assessed in vitro.

## Materials and methods

### Bacterial strains, culture, and growth conditions

Four Gram-negative bacteria including *Salmonella enterica* serovar Typhimurium (*S*. Typhimurium) ATCC 13311 and 16 drug-resistant *S*. *enterica* isolates (*S*. Typhimurium and monophasic variant 4,5,12:i:-), and four Gram-positive bacteria (*Staphylococcus aureus* ATCC 25923, *Staphylococcus epidermidis* ATCC 12228, *Bacillus cereus* ATCC 11778, and *Listeria monocytogenes* 10403s) were used as test microorganisms. The drug-resistant strains previously isolated for genotypic characterization in 2014^21^ were provided by Dr. Soraya Chaturongakul (Mahidol University, Thailand). All bacterial strains were cultured in a proper medium at 37 °C for 18 h (Table 1).

**Table 1:** Minimum inhibitory concentration (MIC) and minimum bactericidal concentration (MBC) of acidocin J1132β derivatives against 9 strains of pathogenic bacteria.

Pathogenic bacteria	Culture medium	MIC (MBC) (µg/ml)					
		A0	A4	A6	A9	A11	Melittin
Gram-negative bacteria							
* Salmonella* Typhimurium ATCC 13311	TSB	> 250 (> 250)	31.25 (31.25)	62.5 (62.5)	62.5 (62.5)	15.63 (31.25)	3.91 (3.91)
* Pseudomonas aeruginosa* ATCC 27853	TSB	> 250 (> 250)	250 (250)	125 (250)	250 (250)	31.25 (62.5)	15.63 (15.63)
* Shigella sonnei* ATCC11060	TSB	> 250 (> 250)	31.25 (31.25)	62.5 (125)	62.5 (125)	31.25 (62.5)	3.91 (7.81)
* Acinetobactero baumanii* MT strain	TSB	> 250 (> 250)	15.63 (31.25)	31.25 (31.25)	62.5 (125)	15.63 (31.25)	7.81 (7.81)
GM^a^ (Gr.- strains)	–	> 250	44.2	62.5	88.39	22.1	6.8
Gram-positive bacteria							
* Staphylococcus aureus* ATCC 25923	TSB	> 250 (> 250)	> 250 (> 250)	> 250 (> 250)	> 250 (> 250)	> 250 (> 250)	3.91 (3.91)
* Staphylococcus epidermidis* ATCC 12228	TSB	> 250 (> 250)	250 (250)	> 250 (> 250)	62.5 (> 250)	15.63 (31.25)	3.91 (3.91)
* Bacillus cereus* ATCC 11778	TSB	> 250 (> 250)	> 250 (> 250)	> 250 (> 250)	> 250 (> 250)	250 (250)	3.91 (7.81)
* Listeria monocytogenes* 10403s	TSBYE	> 250 (> 250)	250 (> 250)	250 (> 250)	250 (> 250)	125 (125)	3.91 (3.91)
GM^b^ (Gr. + strains)	–	> 250	> 250	> 250	125	78.75	3.91
MHC^c^ (µg/ml)	–	–	> 250	> 250	> 250	> 250	˂ 0.98
TI^d^ (Gr.- strains)	–	–	11.31	8	5.66	22.62	0.07
TI^e^ (Gr. + strains)	–	–	1.00	1.00	4.00	6.35	0.13

*TSB* tryptic soy broth, *TSBYE* tryptic soy broth supplemented with 0.6% yeast extract.

^a^GM (Gr.− strains) denotes the geometric mean of MIC values from all Gram-negative strains.

^b^GM (Gr.+ strains) denotes the geometric mean of MIC values from all Gram-positive strains.

^c^MHC is the minimum concentration that caused 10% hemolysis of human red blood cells (hRBC). When no 10% hemolysis, as well as > 10% hemolysis, were observed at 250 µg/ml and 0.98 µg/ml, values of 500 µg/ml and 0.49 µg/ml were used to calculate the therapeutic index, respectively.

^d^Therapeutic index (Gr.− strains) is the ratio of the MHC to the geometric mean of MICs from Gram-negative strains.

^e^Therapeutic index (Gr.+ strains) is the ratio of the MHC to the geometric mean of MICs from all Gram-positive strains.

### Peptide design and sequence analysis

**Figure 1:**
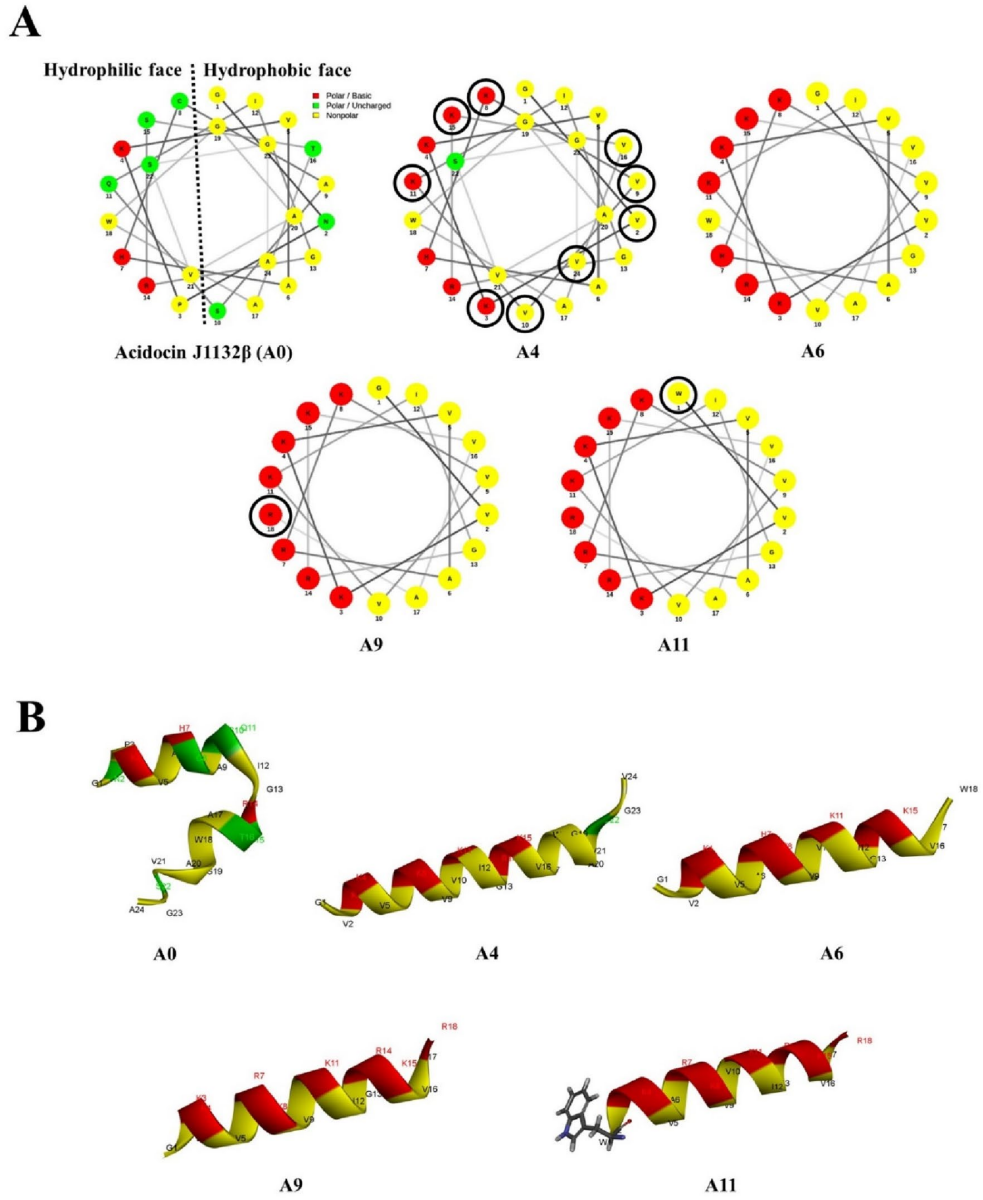
Helical wheel projections (**A**) and three-dimensional structure prediction (**B**) of designed acidocin J1132β peptide derivatives shown in ribbon diagrams. Positively charged, uncharged or polar, and hydrophobic residues are displayed in red, green, and yellow, respectively. The numbers depict the amino acid position.

The amino acid sequence of acidocin J1132β (A0 peptide) was retrieved from UniProtKB (https://www.uniprot.org/uniprot/Q9R499) and utilized as a template to design a new series of peptide derivatives by substitution with polar positively charged and nonpolar hydrophobic amino acids, and truncation strategies. Based on a helical wheel projection of the A0 parent peptide, it was discovered that the polar (left) face was separated from the hydrophobic (right) face (Fig. 1), which was advantageous for amino acid substitution. A4 was designed by substituting with lysine (K) in the polar face and valine (V) in the hydrophobic face of A0 at positions 2, 3, 8, 9, 10, 11, 15, 16 and 24 (V2N, K3P, K8C, V9A, V10S, K11Q, K15S, V16T and V24A). A6 was a form of A4 truncated by removing a 6-amino acid unstructured region at the C terminus (GAVSGV) (Fig. 1). The amphipathic A9 was sequentially modified from A6 by replacing with two arginines on the polar face at positions 7 and 18 (R7H and R18W). A11, the peptide with the highest positive charge and most hydrophobicity, was serially modified by replacing glycine in A9 peptide with tryptophan (W1G) at the polar/nonpolar interface. All derivatives (Table 2) were further modified by post-translational amidation at the C terminus. The physicochemical properties of all designed peptides were analyzed using Antimicrobial Peptide Calculator and Predictor (APD3 Server: https://aps.unmc.edu/prediction). Peptide 3D structure models were predicted by I-TASSER (https://zhanggroup.org/I-TASSER/) and helical wheel projections were predicted using the online program NetWheels (http://lbqp.unb.br/NetWheels/).

**Table 2:** Amino acid sequence and physicochemical properties of acidocin J1132β derivatives.

Peptide	Sequence	Theoretical MW	Measured MW^a^	aa^b^	Net charge	Pho%^c^	µH^d^
A0	GNPKVAHCASQIGRSTAWGAVSGA-NH_2_	2325.59	2323.62	24	+ 2.25	42	0.065
A4	GVKKVAHKVVKIGRKVAWGAVSGV-NH_2_	2474.04	2473.08	24	+ 6.25	50	0.315
A6	GVKKVAHKVVKIGRKVAW-NH_2_	2003.51	2002.55	18	+ 6.25	50	0.369
A9	GVKKVARKVVKIGRKVAR-NH_2_	1992.53	1991.57	18	+ 8.00	44	0.607
A11	WVKKVARKVVKIGRKVAR-NH_2_	2121.69	2120.73	18	+ 8.00	50	0.637

^a^*MW* molecular weight (g/mol) measured by mass spectroscopy (MS).

^b^aa, number of amino acids.

^c^Pho%, the percentage of hydrophobic residues.

^d^µH, the mean hydrophobic moment determined at website: http://heliquest.ipmc.cnrs.fr/.

### Peptide synthesis

All peptide derivatives, along with tetramethylrhodamine (TAMRA)-labeled A11, were chemically synthesized using a solid-phase method with N-(9-fluorenly) methoxycarbonyl (Fmoc) chemistry and purified by reversed-phase high-performance liquid chromatography (RP-HPLC) (ChinaPeptides, China). The mass of each peptide was analyzed and determined by electrospray ionization mass spectrometry (ESI–MS); all peptides studied, including original and modified derivatives, were diluted in sterile deionized water (DI) to obtain stock solutions of 10 mg/ml and stored at − 20 °C until use.

### Determination of antimicrobial activity

The minimum inhibitory concentration (MIC) of each peptide was evaluated by broth microdilution assay according to a modification described by the National Committee for Clinical Laboratory Standards (NCCLS)^22^. Mid-logarithmic phase bacterial cells were adjusted to an OD_620_ of 0.05 in Mueller–Hinton broth (MHB), a universal medium for antibacterial susceptibility testing^23^. Then, 50 µl of bacterial solution was incubated with 50 µl of two-fold serially diluted peptide (0.98–250 µg/ml in PBS) at 37 °C for 24 h with continuous shaking. MIC values were defined as the lowest concentration of antimicrobial agents that inhibited bacterial growth or showed no turbidity by visible inspection^24^. Bacteria inoculated with melittin, known to kill both Gram-positive and -negative bacteria, and uninoculated MHB were used as positive and negative controls, respectively. The minimal bactericidal concentration (MBC) of each peptide was determined using a colony count assay. Fifty microliter aliquots of the mixture from each non-turbid well (no growth) from the MIC experiment was spread on Tryptic soy agar (TSA) and cultured overnight at 37 °C. MBC values were defined as the lowest concentration of antimicrobial agents where there was no bacterial colony growth on the agar^24^. All tests were done in triplicate.

### Hemolysis assay

The toxicity of all peptide derivatives toward human red blood cells (hRBCs) was investigated by evaluating the amount of hemoglobin released after exposure to peptide^17^. Briefly, hRBCs (blood group O) were collected from a healthy adult volunteer into a heparin-coated polycarbonate tube. The sample was centrifuged for 5 min at 1000g to separate erythrocytes from buffy coat and plasma. The erythrocytes were washed at least three times with 1 × PBS and centrifuged for 5 min at 1000g or until supernatant was clear. The sample was resuspended in PBS to obtain 2% (v/v) hRBCs. To determine hRBC lysis, hRBC suspensions were incubated with equal volumes of various concentrations of peptide (0.98–250 µg/ml) for 1 h at 37 °C. Intact hRBCs were centrifuged for 5 min at 1000g, supernatants were transferred into new 96-well plates, and absorbance was measured at OD 405 nm. Untreated hRBCs (in PBS only) served as negative control (OD_Negative_) and hRBCs treated with 0.1% Triton X-100 as positive control (OD_TritonX-100_). Absorbance of samples was converted to percentage hemolysis according to the equation:

$${\text{Hemolysis }}\left( \% \right) = \left[ {\left( {{\text{OD}}_{{{\text{Sample}}}} {-}{\text{OD}}_{{{\text{Negative}}}} } \right)/\left( {{\text{OD}}_{{{\text{Triton X}} - {1}00}} {-}{\text{ OD}}_{{{\text{Negative}}}} } \right)} \right] \, \times {1}00$$

The experiments involving human participants were performed in accord with the ethical standards of and approved by the Ethics Committee of Thammasat University (COA No. 066/2562). Individual written informed consent was obtained from all participants.

### Time-kill kinetic assay

Bacterial killing kinetics of A11 were investigated against *S*. Typhimurium as described previously^25^. *S*. Typhimurium ATCC13311 was grown in TSB at 37 °C until reaching the exponential phase. Bacterial suspensions were diluted to an inoculum of 10^7^ CFU/ml in MHB and incubated with A11 at a final concentration of 1 × MIC with continuous shaking at 37 °C. The mixture was sampled at 1, 2, 4, 6, 8, 12 and 24 h, and subsequently tenfold serially diluted in 1 × PBS and plated in triplicate on Mueller–Hinton agar (MHA). After incubation at 37 °C for 24 h, the colonies were counted and CFU/ml was calculated. The tests were done in three independent experiments.

### In vitro cytotoxicity determination

The cytotoxicity of A11 was evaluated by the 3-(4,5 dimethylthiazol-2-yl)-2,5-diphenyltetrazolium bromide (MTT) assay^17^. L929 mouse fibroblast cells were cultured in Dulbecco's Modified Eagle Medium (DMEM) supplemented with 1% (v/v) of 100 IU/ml penicillin/streptomycin and 10% (v/v) fetal bovine serum (FBS). The cells were maintained in an air atmosphere containing the 5% CO_2_ under high humidity at 37 °C. L929 cell lines were seeded into a sterile 96-well plate (1 × 10^4^ cells/well) then incubated overnight and treated with different concentrations of A11 (0.98–250 µg/ml) for 24 h. Media were removed, and cultured cells were incubated with 100 µl of MTT solution (4 mg/ml) for 4 h at 37 °C. After removal of MTT solution, the formed formazan was dissolved in 100 µl DMSO. Absorbance was measured by microplate reader at 570 nm; untreated cells served as negative control and melittin-treated cells as a positive control. Cell viability was determined following this equation:

$${\text{Cell viability}}\left( \% \right) = \left( {{\text{OD}}_{{{\text{Treated}}}} /{\text{OD}}_{{{\text{Control}}}} } \right) \times {1}00$$

### Circular dichroism (CD) analysis

To observe the conformational change of A11 induced by membrane-mimicking environmental conditions, the secondary structure of A11 peptide was investigated by CD analysis using a Jasco-815 spectropolarimeter (Jasco, Japan). Peptide solutions were diluted to a final concentration of 150 µM in different environments: deionized water (aqueous environment), 30 mM sodium dodecyl sulfate (SDS; mimicking the negative charge of prokaryotic membranes), and 50% (vol/vol) TFE (2,2,2-trifluoroethanol; mimicking the hydrophobicity of bacterial membranes). CD spectra were recorded at 10 nm/min scanning speed in the spectral range of 190 to 250 nm, with a 0.1-mm-path length quartz cell at room temperature. Three scans were performed for each environment. The acquired CD spectrum was converted to the mean residue ellipticity using the following equation:

$$\theta_{{\text{M}}} = \left( {\theta_{{{\text{obs}}}} /{1}0} \right) \times ({\text{M}}_{{{\text{RW}}}} /{\text{c}} \times {1})$$

where θ_M_ is residue ellipticity (deg./M/m), θ_obs_ is observed ellipticity corrected for the buffer at a given wavelength (mdeg), M_RW_ is residue molecular weight (M_W_/number of amino acids), c is concentration of peptide (mg/ml), and 1 is the path length (cm).

### Membrane-penetrating activity

The interaction of A11 with *S*. Typhimurium cell membrane at various times was observed using TAMRA-labelled A11^18^. *S*. Typhimurium ATCC 13311 were cultured overnight in TSB at 37 °C, washed and diluted to approximately 10^7^ CFU/ml with 1 × PBS. Bacterial suspensions were cultured with TAMRA-labelled A11 at 1 × MIC for 1, 2, 4, 8, 12 or 24 h at 37 °C with shaking. Then, unbound labelled peptides were removed by washing with PBS and centrifuging at 10000g for 10 min at 4 °C. The treated bacterial cells were then collected, resuspended into 1.5 ml of PBS, analyzed by flow cytometry and the orange—red fluorescence signal (585/42 nm) emitted by TAMRA dye determined (CytoFlex, Beckman Coulter, United States). Twenty-five thousand bacterial cells were counted in each sample; tests were repeated three times and the results were examined by Kaluza software (Beckman Coulter, United States).

### Membrane depolarization and permeability

To observe the bacterial membrane lytic mode of A11, propidium iodide (PI) or membrane-impermeant nucleic acid intercalator and bis-(1,3-dibutylbarbituric acid) trimethine-oxonol (BOX) or membrane potential-sensitive dye were used to determine the permeabilization and depolarization of the membrane, respectively^26^. Fluorescence signals corresponded with the loss of bacterial membrane integrity and potential. *S*. Typhimurium ATCC 13311 were cultured overnight in TSB at 37 °C, washed and diluted to approximately 10^7^ CFU/ml with 1 × PBS. The bacterial suspensions were then cultured with A11 at a final concentration of 1 × MIC and incubated for 1, 2, 4, 8, 12 or 24 h with continuous shaking at 37 °C. Unbound peptide was removed by washing with PBS and centrifuging for 10 min at 10,000g. BOX at a final concentration of 0.25 µM (Sigma, Germany) and PI at a final concentration of 7.5 µg/ml (Sigma, Germany) were added to samples. Untreated bacteria or no peptide served as negative controls, while the bacterial cells heated at 70 °C for 30 min served as positive controls in these experiments. The results were collected by flow cytometer (CytoFlex, Beckman Coulter, United States) at a laser excitation wavelength (488 nm). The forward scatter (FS), side scatter (SS), red fluorescence (585/42 nm) emitted by Pl and green fluorescence (530/30 nm) from BOX were recorded using logarithmic scales. Twenty-five thousand cells, determined according to their scatter parameters, were recorded in each test. All tests were done in three independent experiments and the results were analyzed using Kaluza software (Beckman Coulter, United States).

### Transmission electron microscopy (TEM)

The intracellular alterations and changes in structural integrity of *S.* Typhimurium treated with A11 were investigated under TEM^27^. *S.* Typhimurium ATCC 13311 were cultured overnight in TSB at 37 °C, diluted to an OD_620_ of 0.2 and incubated with 0.5 × MIC of A11 for 4 or 12 h with continuous shaking at 37 °C. The bacterial suspensions were fixed with 2.5% glutaraldehyde for 24 h. Cell pellets were washed and rinsed in PBS, then post-fixed for 2 h in 1% osmium tetroxide with PBS. The fixed bacterial cells were washed twice in PBS and dehydrated in a series of graded ethanol (50%, 70%, 90% and 100%) for 15 min, then soaked with absolute acetone for 20 min. Samples were transferred into a solution of epoxy resin (1:3) and lute acetone (1:1). After 60 min, the sample was transferred into pure epoxy resin and left overnight. Ultrathin section was cut on an ultramicrotome with a glass knife, and post-stained with lead citrate and uranyl acetate, then investigated using a transmission electron microscope (Hitachi HT7700, Japan).

### DNA binding assay

The ability of A11 to bind DNA was investigated using a gel retardation method^28^. The overnight cultures of *S.* Typhimurium ATCC 13311 in TSB at 37 °C were collected by centrifugation at 4000g for 10 min; the cell pellets were used to extract genomic DNA by E.Z.N.A. Bacterial DNA Kit (Omega Biotek, United States). Then, 400 ng of genomic DNA was mixed with twofold serially diluted concentrations (0.98–250 μg/ml) of A11 peptide in binding buffer [binding buffer: 1 mM ethylene diamine tetraacetic acid (EDTA), 10 mM Tris–HCl (pH 8.0), 20 mM KCl, 1 mM dithiothreitol (DTT), 5% glycerol, and 50 µg/ml BSA]. After incubation at 37 °C for 1 h, binding was assessed by 1% agarose gel electrophoresis.

### Effect of pH

The pH of peptide solutions was adjusted (pH 3, 5, 7, 9 and 11) using 1 M acetic acid (CH_3_COOH) and 1 M sodium hydroxide (NaOH). Solutions adjusted to the different pHs without addition of A11 were used as controls. Subsequently, twofold serially diluted solutions (0.98–250 μg/ml) of A11 peptide were prepared with different pHs of peptide solution and incubated at 37 °C for 60 min, transferred to 1 × 10^7^ CFU/ml of *S.* Typhimurium ATCC 13311 suspensions, then incubated at 37 °C with continuous shaking. After 24 h, MIC values were determined.

### Effect of temperature

Two-fold serial dilutions (0.98–250 μg/ml) of A11 peptide were prepared and pre-incubated for 60 min at one of various temperatures (40 °C, 60 °C, 80 °C and 100 °C), and then cooled to 4 °C. After 30 min, 50 μl of heated peptide solutions were cultured with 50 μl of *S.* Typhimurium ATCC 13311 suspensions (approximately 1 × 10^7^ CFU/ml). After 24 h incubation at 37 °C, MICs were determined; all experiments were done in triplicate.

### Effect of salt concentration

Two-fold serial dilutions (0.98–250 μg/ml) of A11 with various concentrations of MgCl_2_ and NaCl (1 mM MgCl_2_, 5 mM MgCl_2_, 1% NaCl, 3% NaCl, 5% NaCl and 10% NaCl) were prepared and incubated at 37 °C for 60 min. Then, 50 μl of A11 in different salt concentrations were incubated with 50 μl of *S.* Typhimurium ATCC 13311 suspension (approximately 1 × 10^7^ CFU/ml) at 37 °C with shaking. After 24 h, MICs were determined; all experiments were done in triplicate.

### Combination effect of candidate peptide with nisin

Effects of the combination of A11 with nisin against *S*. Typhimurium ATCC 13311 and 10 drug-resistant *S.* Typhimurium and *S. enterica* isolates were determined by the checkerboard titration method^29^. Briefly, 10 drug-resistant *S.* Typhimurium were grown to a concentration of 1 × 10^7^ CFU/ml in fresh TSB. Aliquots (25 µl) of nisin (final concentrations of 0.5–16 mg/ml) and A11 (final concentrations of 0.98–250 µg/ml) were added into 96-well plates containing 50 µl of bacterial suspension and cultured at 37 °C. After 24 h of incubation, the MIC values were observed and the combined effects of A11 with nisin were calculated as a combination index (CI) to analyze drug-drug interaction using the following equation:

$$\text{CI} = \frac{{\text{C}}_{\text{A}}}{{\text{IC}}_{\text{A}}}\text{+}\frac{{\text{C}}_{\text{B}}}{{\text{IC}}_{\text{B}}}$$

where C_A_ is the concentration of compound A contained in a combination that provides the desirable effect, C_B_ is the concentration of compound B contained in a combination that provides the desirable effect. IC_A_ is the concentration required to produce the same effect as compound A when used alone, IC_B_ is the concentration required to produce the same effect as compound B when used alone. The CI results were interpreted as follows: a CI of less than 1 indicates synergistic effect, a CI equal to 1 indicates additive effect, and a CI of more than 1 indicates antagonistic effect^30^.

### Statistical analysis

All experiments were done in triplicate and results are displayed as mean ± standard deviation (SD). The differences between treated and control groups were analyzed by ANOVA with Tukey's Post Hoc Test using GraphPad PRISM software (version 7.0, GraphPad Software, United States). In all analyses, differences were considered statistically significant at the 95% confidence level (*p* < 0.05).

## Results

### Peptide design and sequence analysis

In this study, four peptide derivatives (A4, A6, A9 and A11) were sequentially designed from the sequence of the parent peptide, acidocin J1132β (A0), by substitution with amino acid and truncation strategies. The type and position of amino acid residues for substitution depended on polar or nonpolar faces of targeted residues on a helical wheel projection. The hydrophobicity, net positive charge, amphipathic conformation and secondary structure of peptide were considered to be potentially crucial factors for antibacterial activity^31,32^. Therefore, derivatives were designed to increase in total net charge, hydrophobicity and amphipathicity (µH). All designed acidocin derivatives were high in basic amino acids (K and R) as well as hydrophobic valine, thus conferring net charge ranging from + 6.25 to + 8.00, and possession of 44–50% hydrophobic residues which may promote amphipathic conformations upon interaction with bacterial membranes (Table 2).

Sequentially designed, the substitution with lysine on polar faces and valine on nonpolar faces in the 24-amino acid A0 peptide produced the A4 peptide with increases of both net positive charge (+ 2.25 to + 6.25) and hydrophobicity (42–50%). The 18-residue A6 peptide, an N-terminal truncated form of A4, displayed similar net charge and hydrophobicity with A4, but had a shorter amino acid sequence. To further enhance overall net charge, A9 was derived by replacing two arginines on hydrophilic faces, resulting in an increase of positive charge from + 6.25 to + 8.00. Because tryptophan at position 24 was substituted with positively charged arginine, the hydrophobicity of A9 was reduced from 50 to 44%, as the hydrophobic/hydrophilic interface serves as a crucial site for antibacterial activity^16,33^. A11 was designed as a modified cation from the A9 amphipathic peptide, substituting glycine with tryptophan at position 1. Among all acidocin J1132β derivatives, the novel 18-residue A11 peptide displayed the highest positive charge (+ 8.00), hydrophobicity (50%) and mean hydrophobic moment (0.637), along with a perfect amphipathic helical structure (Fig. 1). The molecular weights of all peptide derivatives were verified by ESI–MS. They were in accordance with the theoretical values demonstrating that the peptide synthesis was successful (Table 2).

### Antimicrobial and hemolytic activities

Antibacterial activity and hemolysis caused by acidocin J1132β derivatives were employed as screening and prerequisite tests for choosing a candidate peptide. The MIC and MBC values of the peptides against the panel of human pathogenic bacterial strains, and that of the positive control peptide (melittin), are shown in Table 1. The parental acidocin J1132β peptide, A0, had no antibacterial activity against any of the tested bacteria even at maximum concentration (MIC > 250 μg/ml). Remarkably, the modified A4 peptide and its truncated form, A6, showed higher antibacterial activity than did A0, especially against Gram-negative bacteria with geometric mean (GM) values of 44.2 and 62.5, respectively. The two arginine substituted peptide, A9, demonstrated broad-spectrum antibacterial activity with MICs ranging from 62.5 to 250 μg/ml. Among all derivatives, A11 peptide showed the most active antimicrobial effect against both Gram-negatives [especially *Salmonella enterica* serovar Typhimurium (*S*. Typhimurium) and *Acenitobacter baumanii*] and Gram-positives (especially *Staphylococcus epidermidis*). Melittin (known as membrane-lytic peptide derived from bee venom) served as a positive control, showing potent broad-spectrum antibacterial activity with low GM values.

**Figure 2:**
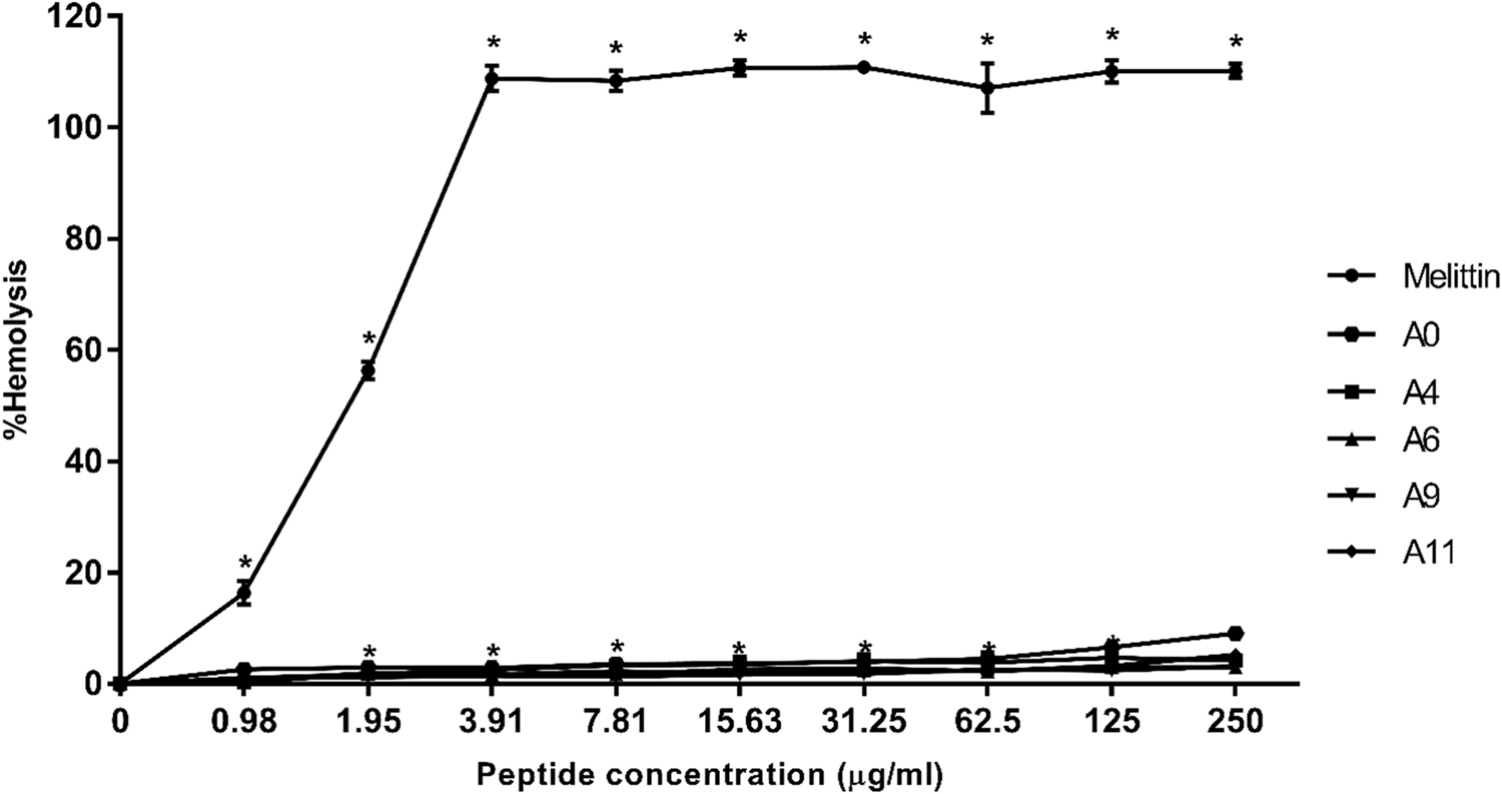
Hemolytic activity of A11 peptide and melittin against human red blood cells (hRBCs). The experiments were done in triplicate and the data are represented as the mean ± SD. The statistical analysis was performed using two-way ANOVA and Tukey’s test (p < 0.05). An asterisk (*) indicates a statistically significant difference when compared to that of the negative control.

The hemolytic activity of acidocin J1132β derivatives toward human RBCs was evaluated at various concentrations (0.98–250 µg/ml) (Fig. 2). A control peptide, melittin, induced complete lysis of human RBCs at a concentration of 3.91 μg/ml. The parent A0 peptide and its derivatives exhibited no obvious hemolytic effect (less than 6% hemolysis) even at the maximum concentration (250 µg/ml). The hemolytic activity of the most active peptide derivative (A11) at the MIC concentration was less than 2%. Additionally, the therapeutic index (TI) of each peptide was evaluated as a quantitative measurement of the peptide’s relative safety. The TI was defined as the ratio between the geometric mean of MIC and the MHC, the minimum concentration that caused 10% hemolysis. The higher the TI, the more specificity the peptide had for bacterial- than mammalian-like membranes^34^. The melittin showed very narrow therapeutic windows (TI < 0.2), While all designed peptides showed much higher TIs than did melittin. Peptide A11 was the novel antibacterial peptide candidate with widest therapeutic window (Table 1).

The antibacterial activity of A11 against *S*. Typhimurium ATCC 13311 and 16 drug-resistant *S*. Typhimurium and 4,5,12:i:- isolates from Thailand was determined and compared to that of nisin, an approved food preservative (Table 3). A11 displayed potent activity against *S*. Typhimurium with an MIC and MBC of 15.63 and 31.25 µg/ml, respectively, while the MIC of nisin was 16,000 µg/ml. Nisin had no antimicrobial activity against any of the 16 tested drug-resistant *S*. *enterica* isolates (up to maximum concentration of 16,000 µg/ml). In contrast, peptide A11 displayed potent antimicrobial activity against all bacterial strains tested with MICs ranging from 62.5 to 125 μg/ml.

**Table 3:** Minimum inhibitory concentration (MIC) and minimum bactericidal concentration (MBC) of A11 against 16 drug-resistant *Salmonella enterica* strains. All strains were grown in Tryptic Soy Agar (TSA).

Serovar	Strains	Drug susceptibility*		MIC(MBC) in µg/ml	
		Resistance	Intermediate	A11	Nisin
*S*. Typhimurium	ATCC 13311	–	–	15.63 (31.25)	16,000
*S*. Typhimurium	H1-001	Amp, Ctx, C, Cp, NA, S, T	Amc	62.5 (125)	> 16,000
*S*. Typhimurium	H1-006	NA	S, Cp	125 (125)	> 16,000
*S*. Typhimurium	H1-011	Amp, S, T	Amc, Ctx, Cp, NA	62.5 (62.5)	> 16,000
*S*. Typhimurium	H1-015	Amp, S	Cp	62.5 (62.5)	> 16,000
4,5,12:i:-	H1-024	Amc, Amp, Ctx, C, NA, S, SxT	Cp	62.5 (62.5)	> 16,000
*S*. Typhimurium	H1-041	Amp, Ctx, C, S, T, SxT	Amc, Cp, NA	125 (125)	> 16,000
*S*. Typhimurium	H1-062	Amc, Amp, Ctx, C, NA, S, T	Cp	125 (125)	> 16,000
4,5,12:i:-	H1-100	Amp, C, Cp, S, T, SxT	Amc, NA	62.5 (62.5)	> 16,000
4,5,12:i:-	H2-010	Amp, C, S, T	–	62.5 (62.5)	> 16,000
4,5,12:i:-	H2-039	Amp, S, T	–	62.5 (125)	> 16,000
4,5,12:i:-	H2-042	Amp, C, S, T, SxT	Cp	62.5 (125)	> 16,000
4,5,12:i:-	H2-047	Amp, S	Cp	62.5 (125)	> 16,000
4,5,12:i:-	H2-049	Amp, S, T	–	62.5 (125)	> 16,000
4,5,12:i:-	H2-067	Amp, S, T	–	62.5 (62.5)	> 16,000
4,5,12:i:-	H2-071	Amp, Ctx, C, S, T, SxT	Cp	125 (125)	> 16,000
4,5,12:i:-	H2-089	Amp, S	–	62.5 (125)	> 16,000

*Amc* amoxicillin/clavulinic acid, *Amp* ampicillin, *Ctx* cefotaxime, *C* chloramphenicol, *Cp* ciprofloxacin, *NA* nalidixic acid, *Nor* norfloxacin, *S* streptomycin, *T* tetracycline, *SxT* sulfamethoxazole/trimethoprim.

*In vitro drug susceptibility of all isolates was performed using Kirby-Bauer disk diffusion test, in accordance with the standard procedure recommended by the Clinical and Laboratory Standards Institute (CLSI), and interpreted as susceptible, resistant, or intermediate according to interpretative criteria based on CLSI breakpoints^29^. The data were taken from Huoy, Pornruangwong, Pulsrikarn, and Chaturongakul (2014).

### Time-kill kinetics

**Figure 3:**
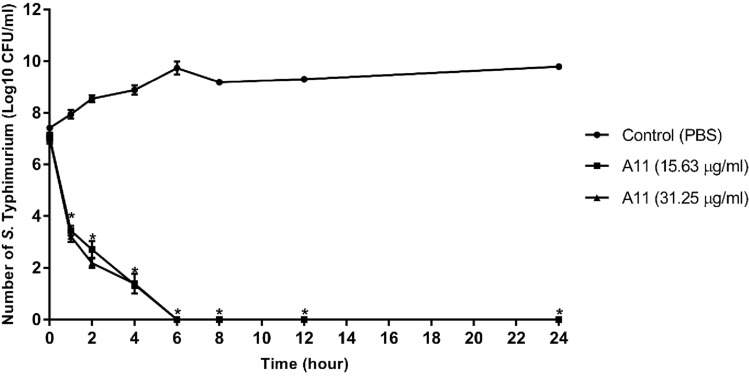
Effect of A11 peptide at MIC and MBC concentrations on the time-kill curve of *S*. Typhimurium ATCC 13311 for 0, 1, 2, 4, 6, 8, 12 and 24 h. The experiments were done in triplicate and the data are represented as the mean ± SD. The statistical analysis was performed using two-way ANOVA and Tukey’s test (p < 0.05). An asterisk (*) indicates a statistically significant difference when compared to that of control group.

The time course of inhibition by A11 toward *S*. Typhimurium was investigated. As shown in Fig. 3, the control group (no peptide) showed a constant increase in growth, whereas [at MIC and MBC (2 × MIC) concentrations] A11 displayed rapid killing in a time-dependent manner. Within 1 h, A11 reduced the concentration of *S*. Typhimurium to approximately 10^3^ CFU/ml at both concentrations. Moreover, A11 completely killed *S*. Typhimurium (more than 99% killing) with no regrowth at MIC nor 2 × MIC concentrations within 6 h.

### Cytotoxicity of peptide

**Figure 4:**
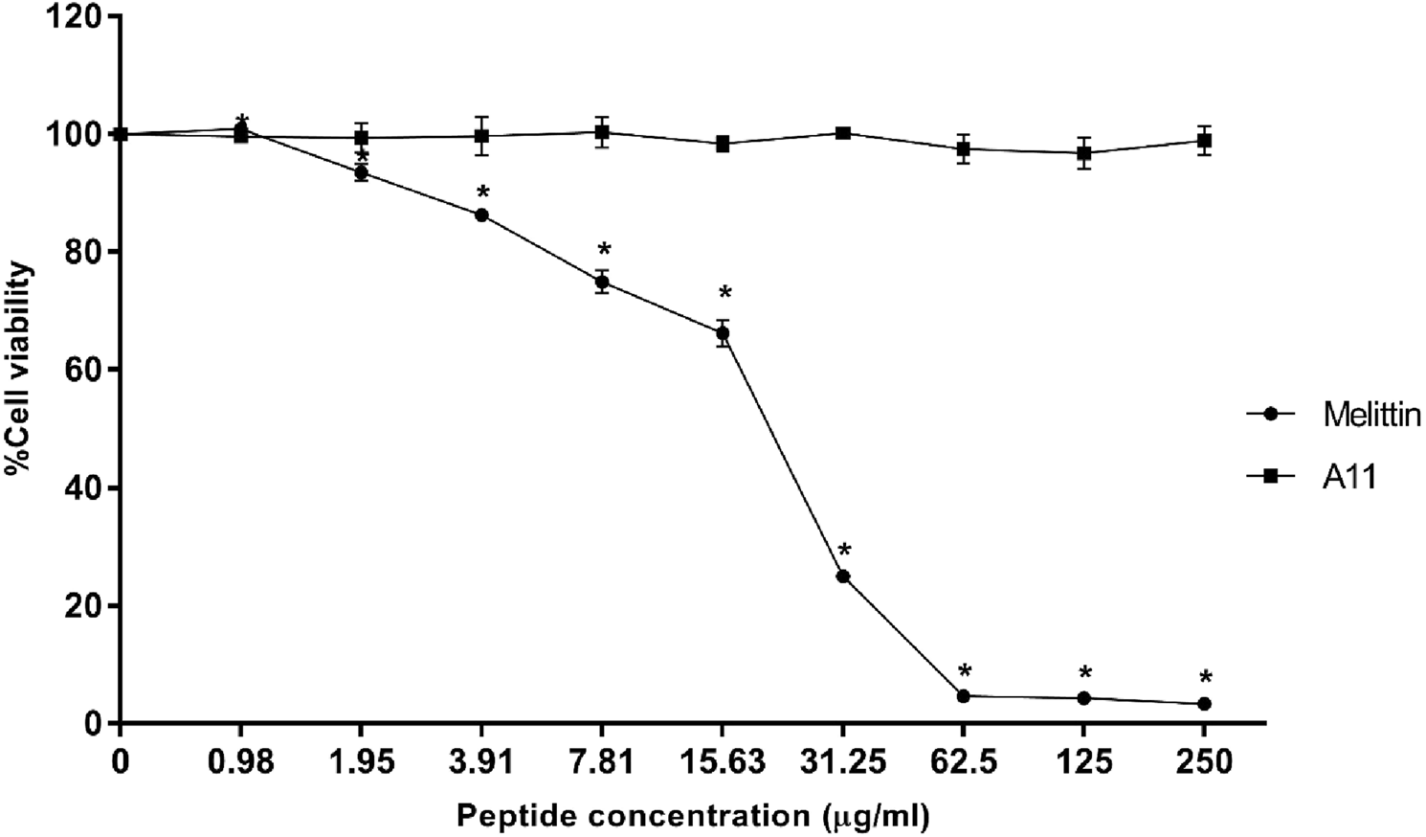
Cytotoxicity of A11 peptide and melittin toward L929 mouse fibroblast cells. The tests were done in triplicate and the results were displayed as the mean and standard deviation. The statistical analysis was carried out using one-way ANOVA and Tukey’s test (p < 0.05). An asterisk (*) indicates a statistically significant difference when compared to that of the negative control.

The cytotoxicity of A11 was investigated using the L929 mouse fibroblast cell in an MTT assay. As shown in Fig. 4, the positive control (melittin) was strongly cytotoxic and dramatically reduced cell viability. A11 exhibited no cytotoxic activity, even at the highest concentration of 250 μg/ml.

### Secondary structure of peptide

**Figure 5:**
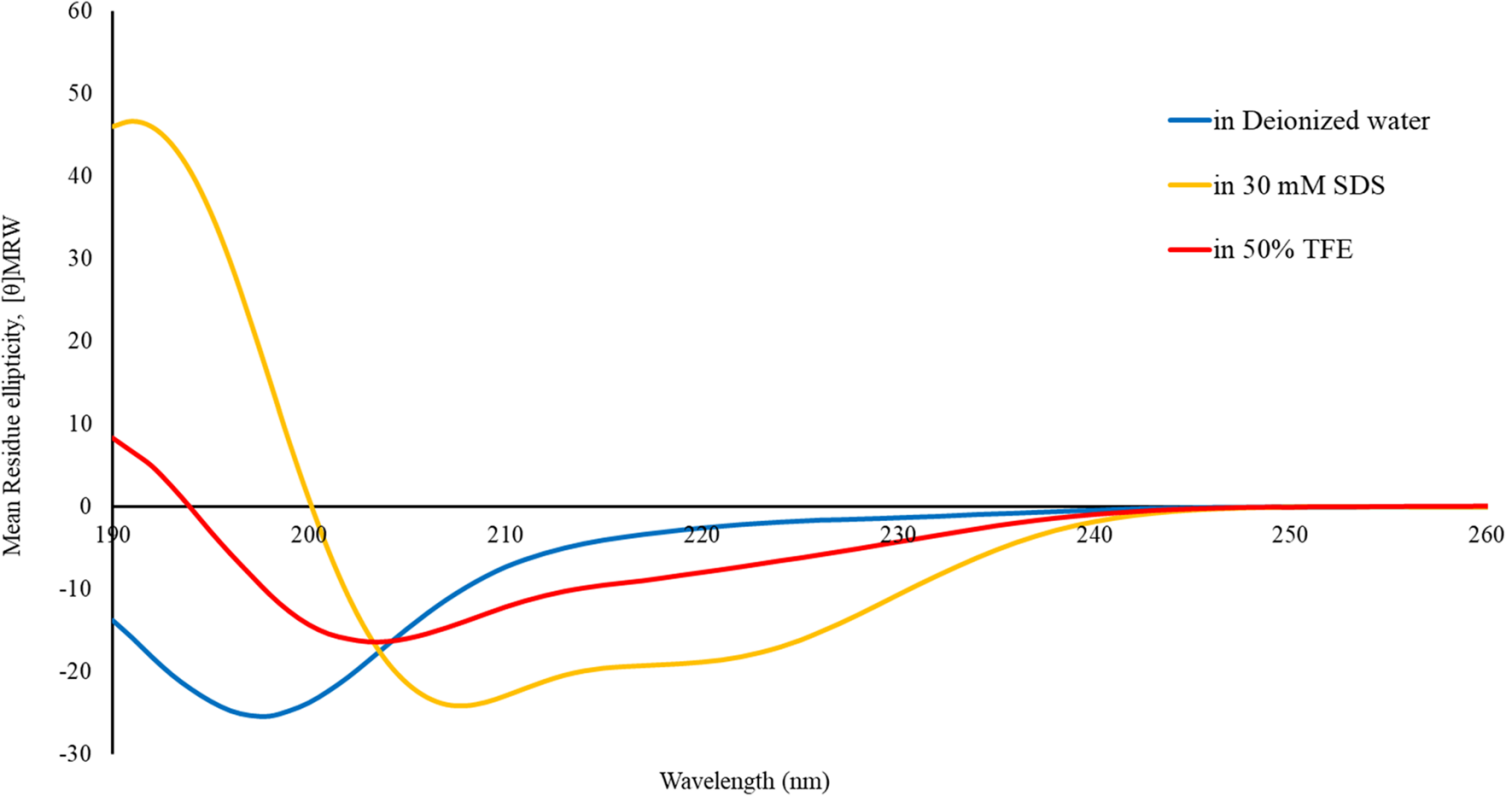
Conformational changes of A11 peptide, evaluated by CD spectroscopy. CD spectra of A11 in aqueous solution (deionized water), 30 mM SDS micelles, and 50% TFE are displayed in blue, yellow, and red, respectively.

The secondary structure of A11 was analyzed by CD spectroscopy to determine the conformational change of the peptide in aqueous (deionized water) and membrane-mimetic conditions (50% TFE, and 30 mM SDS). As shown in Fig. 5, A11 formed a random coil structure in aqueous environments, as demonstrated by the appearance of a negative peak near 200 nm. In 30 mM SDS, the CD spectrum showed one positive peak near 192 nm and two negative dichroic bands at 208 and 222 nm, consistent with the formation of α-helical structures. In 50% TFE, the CD spectrum of A11 revealed a random coil structure. This structural transformation from a random coil to an α‐helix suggested that A11 had a high propensity to form an amphiphilic α-helical secondary structure when the environment was changed from aqueous to negatively charged SDS micelles.

### Membrane-penetrating activity

**Figure 6:**
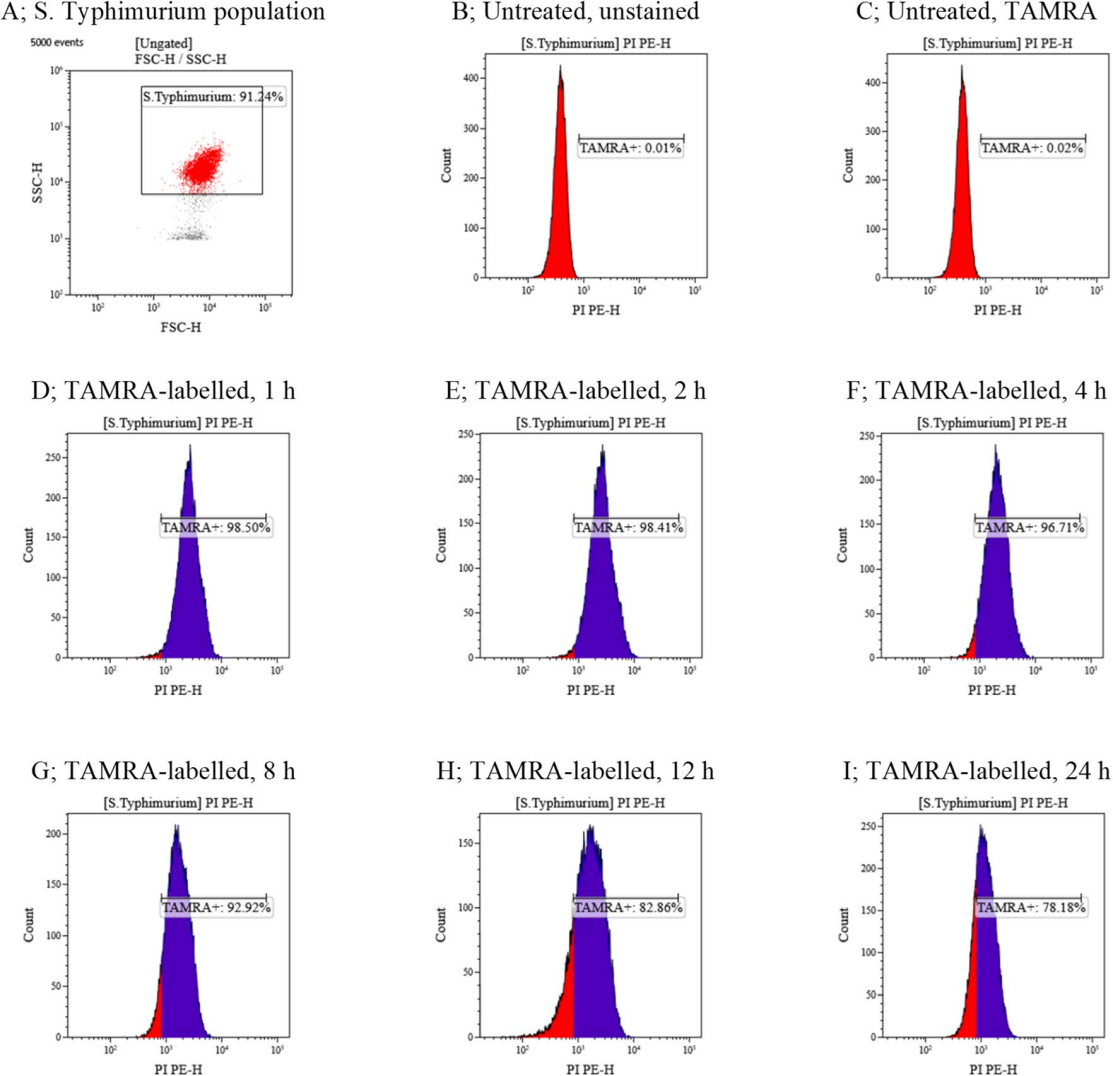
The membrane-penetrating activity of TAMRA-labelled A11 at 1 × MIC to *S*. Typhimurium ATCC 13311 as measured by flow cytometry. *S*. Typhimurium cell population (**A**), and untreated *S*. Typhimurium without staining (**B**). Untreated *S*. Typhimurium stained with TAMRA (**C**), and membrane-penetrating effect of TAMRA-labelled A11 after 1, 2, 4, 8, 12 and 24 h incubation (**D**–**I**).

The interaction of A11 with the membrane of *S*. Typhimurium was observed by flow cytometry using TAMRA-labeled A11. As shown in Fig. 6 and Supplementary Fig. 1, a *S*. Typhimurium population (91.24%) was used for analysis in the present study (Fig. 6A). The control group (no TAMRA-labelled A11) was no TAMRA fluorescent signal (Fig. 6B,C) demonstrating non-autofluorescence and health of the *S*. Typhimurium bacterial cells. TAMRA-labelled A11-treated *S*. Typhimurium cells displayed red fluorescence with 98.50 ± 0.08%, 98.41 ± 0.60%, 96.71 ± 2.46%, 92.92 ± 0.80%, 82.86 ± 6.84% and 78.18 ± 1.59% at 1, 2, 4, 8, 12 and 24 h, respectively (Fig. 6D–I). These results indicated that TAMRA-labelled A11 exhibited binding and penetration through *S*. Typhimurium membranes, and accumulation in cells after 1, 2, 4, 8, 12 and 24 h of incubation.

### Membrane depolarization and permeability

**Figure 7:**
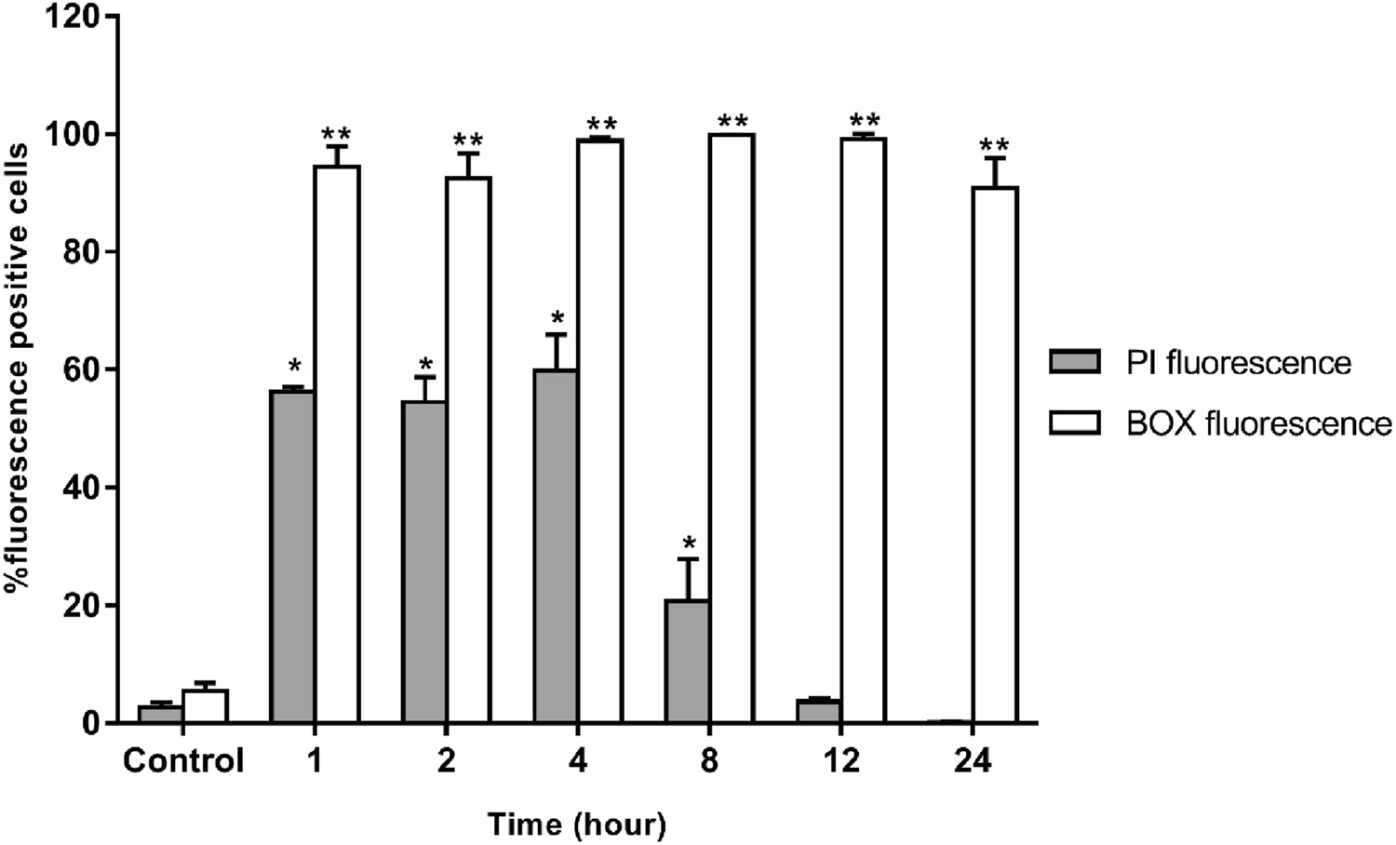
The effect of A11 peptide at 1 × MIC on the *S*. Typhimurium ATCC 13311 membrane permeability (PI) and potential (BOX) after 1, 2, 4, 8, 12 and 24 h of incubation as evaluated by flow cytometry. The tests were assessed in three independent experiments and the results were displayed as the mean and standard deviation. The statistical analysis was performed using two-way ANOVA and Tukey’s test (p < 0.05). An asterisk (*) indicates a statistically significant difference when compared to that of A11-untreated group stained with PI. The double asterisk (**) indicates a statistically significant difference when compared to that of A11-untreated group stained with BOX.

The effect of A11 on cell membranes of *S*. Typhimurium was investigated by flow cytometry. PI fluorescent dye is a cell impermeable, nucleic acid-staining dye which can intercalate in DNA after cell membrane disruption; BOX penetrates depolarized cells and binds to intracellular proteins or membranes and exhibits increased fluorescence. The results are shown in Fig. 7 and Supplementary Fig. 2. In the absence of A11, 92.27 ± 0.97% of *S*. Typhimurium cells exhibited neither PI nor BOX fluorescent signal reflecting intact cell membranes. *S*. Typhimurium cells heated at 70 °C for 30 min served as a positive control. In this study, heat caused high degrees of both permeabilization and depolarization of bacterial membranes (93.52 ± 2.42%). After exposure to A11 at 1 × MIC for 4 h, the *S*. Typhimurium cells displayed a maximum level of membrane permeabilization and depolarization (68.14 ± 5.37%). After 8 h of A11 treatment, a decrease was seen in permeabilization of *S*. Typhimurium. The decrease continued at 12 h, and at 24 h the PI fluorescence signal was not significantly different than that of untreated cells; the BOX fluorescence signal remained high (95.40 ± 3.16%).

### Membrane integrity and intracellular alterations

**Figure 8:**
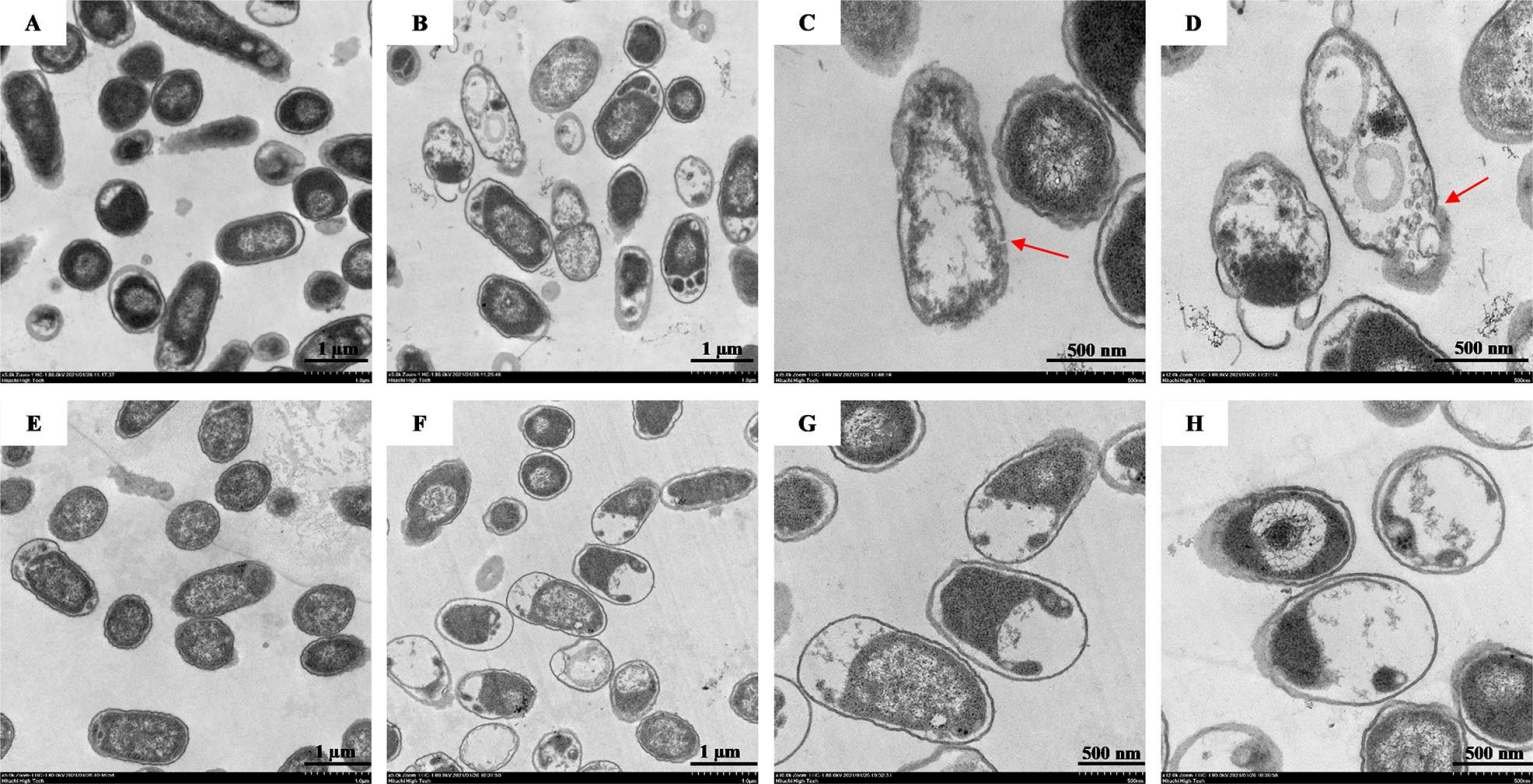
Transmission electron micrographs of *S*. Typhimurium ATCC 13311 exposed to 0.5 × MIC of A11. Control cells without A11 treatment, as a bacterial cell is obtained in 0.2 M PBS buffer for 4 and 12 h (**A** and **E**, respectively). Bacteria treated with A11 for 4 h (**B**–**D**); and bacteria treated with A11 for 12 h (**F**–**H**). Red arrow indicates detectable pore and leakage of intracellular compartments.

Based on flow cytometry, A11 showed time-dependent permeabilization activity which was highest at 4 h and gradually decreased after 12 h of incubation. To further prove the action of A11 on membrane and intracellular contents, *S*. Typhimurium cells were incubated with A11 at 0.5 × MIC for 4 or 12 h. A control group (incubated with 0.2 M PBS) for either 4 or 12 h showed homogenous cytoplasm with intact cell membranes (Fig. 8A,E). When *S*. Typhimurium cells were treated with A11 for 4 h, most cells exhibited morphologic changes (Fig. 8B): disrupted bacterial membranes with detectable pores; release of intracellular compartments as shown by the red arrows (Fig. 8B–D). However, the TEM figures showed no significant change of outer cell membrane morphology after *S*. Typhimurium cells were treated with A11 for 12 h, though most of the cells showed obvious cytoplasmic clearance and appeared as bacterial ghost cells (Fig. 8F–H).

### DNA binding

**Figure 9:**
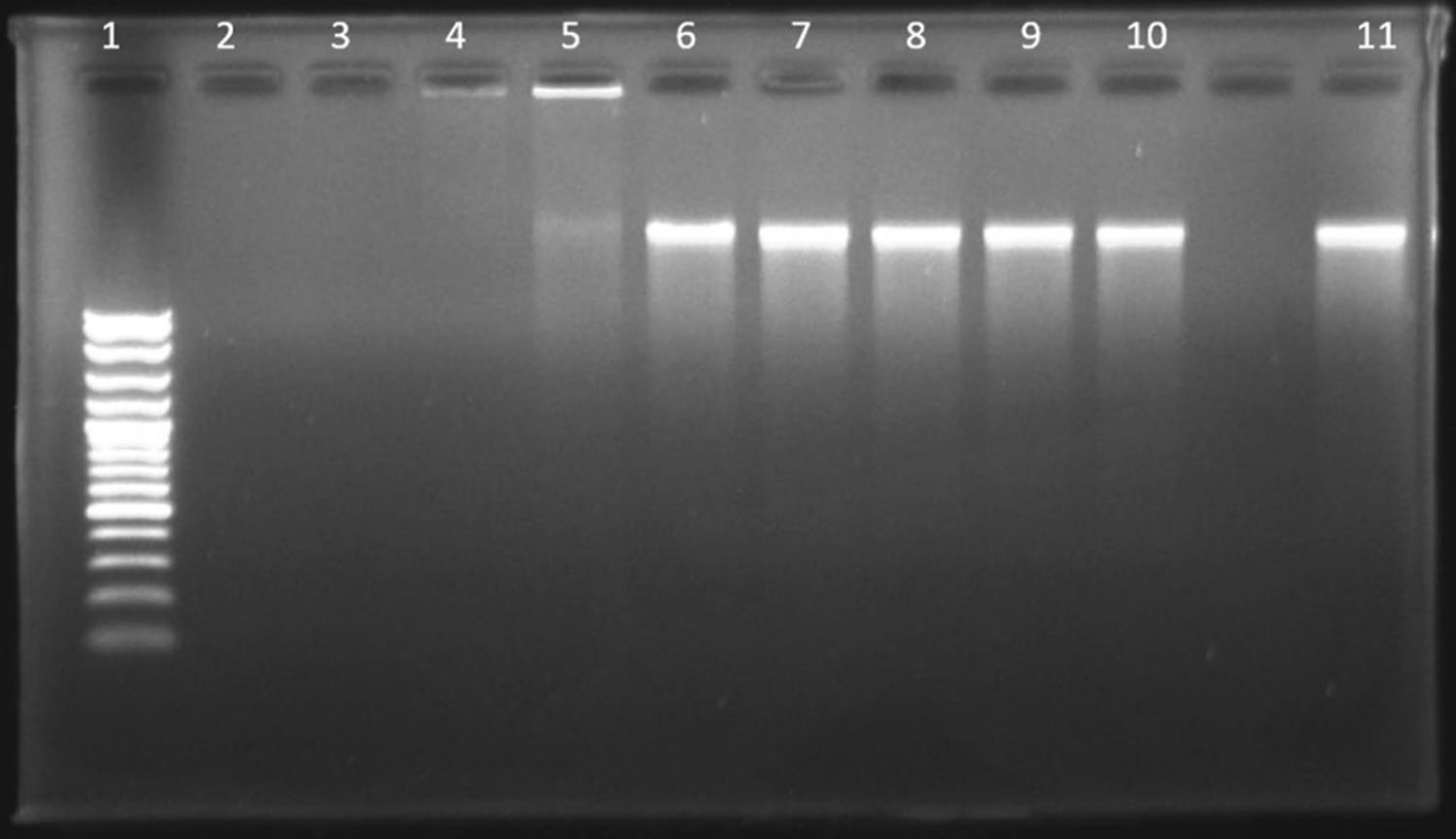
Gel retardation experiment used to measure the binding ability of A11 with genomic DNA of *S*. Typhimurium ATCC 13311. Lane 1: DNA Marker, lane 2: 250 μg/ml A11, lane 3: 125 μg/ml A11, lane 4: 62.5 μg/ml A11, lane 5: 31.25 μg/ml A11, lane 6: 15.63 μg/ml A11, lane 7: 7.81 μg/ml A11, lane 8: 3.91 μg/ml A11, lane 9: 1.95 μg/ml A11, lane 10: 0.98 μg/ml A11; lane 11: plasmid DNA alone.

Some antimicrobial peptides have the ability to kill or inhibit bacterial cells by interacting with intracellular components such as nucleic acids (DNA or RNA), leading to interference with synthesis, replication and/or translation processes^35^. Therefore, different concentrations of A11 were incubated with fixed amounts of *S*. Typhimurium genomic DNA to investigate the DNA-binding ability of A11 (by monitoring shifts with electrophoresis on an agarose gel). As shown in Fig. 9, A11 interacted with bacterial genomic DNA and retarded its migration within agarose gel. At a peptide concentration of 15.63 μg/ml, most genomic bacterial DNA migrated through the gel. When the peptide concentration was increased (31.25 μg/ml), only a small amount of the bacterial DNA was able to migrate through the gel, whereas at a higher concentration of A11 (> 62.5 μg/ml) no migration was observed.

### Effects of pH, temperature and salts

The effects of different pHs, temperatures and salts on the activity of A11 against *S*. Typhimurium are presented in Table 4. The antimicrobial activity of A11 was only two-fold decreased when the pH was between 5 and 11 (MIC of 31.25 μg/ml). At low pH (pH 3), the peptide's antimicrobial activity was dramatically decreased, with MIC value of 125 μg/ml. There was no significant change of the peptide’s activity at pH 7 and 9. After heat treatment of A11 at various temperatures from 40 to 100 °C for 1 h, there was no effect on its antibacterial activity [same MIC value (15.63 μg/ml) at each temperature)]. Regarding different salt concentrations, A11 retained antibacterial activity in the presence of 1 mM MgCl_2_ and 1% NaCl with MIC of 15.63 μg/ml. In 5 mM MgCl_2_, and 3%, 5%, and 10% NaCl, A11 exhibited sensitivity to higher concentrations of both salts with an obvious decrease in antibacterial activity only at the highest concentration of NaCl.

**Table 4:** MIC values (µg/ml) of A11 in the different environmental conditions against *S. enterica* serovar Typhimurium ATCC 13311.

MIC (µg/ml)																
Conditions	Control*	pH					Temperature (°C)				Salt concentrations					
		3	5	7	9	11	40	60	80	100	1 mM MgCl_2_	5 mM MgCl_2_	1% NaCl	3% NaCl	5% NaCl	10% NaCl
A11	15.63	125	31.25	15.63	15.63	31.25	15.63	15.63	15.63	15.63	15.63	31.25	15.63	125	250	> 250

*Control MIC values were determined in the absence of those different environmental conditions.

### Interactions of peptide with nisin

Interactions between A11 peptide and nisin against *S*. Typhimurium ATCC 13311 and 10 drug-resistant *S.* Typhimurium and *S. enterica* isolates were determined in vitro using a checkerboard assay. As summarized in Table 5, A11 combined with nisin showed synergistic interactions toward all strains of *S*. Typhimurium and their monophasic variants including standard strain ATCC13311 and 10 drug-resistant *S.* Typhimurium and *S. enterica* isolates. In the synergistic inhibitory effects on tested bacteria, A11 not only reduced the MIC of nisin (from more than 16–0.5 mg/ml), but also decreased two- to four-fold the MIC of A11 (from 62.5–125 μg/ml to 15.63–31.25 μg/ml).

**Table 5:** In vitro interaction between A11 peptide and nisin against 10 drug-resistant *Salmonella enterica* strains.

Strains		MIC (µg/ml)				CI	Interpretation
		MIC (alone)		MIC (combination)			
		A11	Nisin	A11	Nisin		
*S*. Typhimurium	ATCC13311	15.63	16,000	3.91	4000	0.500	Synergy
*S.* Typhimurium	H1-001	62.5	> 16,000	31.25	500	0.516	Synergy
*S*. Typhimurium	H1-006	125	> 16,000	31.25	500	0.266	Synergy
*S*. Typhimurium	H1-011	62.5	> 16,000	31.25	500	0.516	Synergy
*S*. Typhimurium	H1-015	62.5	> 16,000	31.25	500	0.516	Synergy
*S*. Typhimurium	H1-041	125	> 16,000	31.25	500	0.266	Synergy
*S*. Typhimurium	H1-062	125	> 16,000	31.25	500	0.266	Synergy
4,5,12:i:-	H1-100	62.5	> 16,000	31.25	500	0.516	Synergy
4,5,12:i:-	H2-039	62.5	> 16,000	31.25	500	0.516	Synergy
4,5,12:i:-	H2-042	62.5	> 16,000	15.63	500	0.266	Synergy
4,5,12:i:-	H2-089	62.5	> 16,000	15.63	500	0.266	Synergy

At MIC value of > 16,000 µg/ml, a value of 32,000 µg/ml was used to calculate the combination index (CI).

## Discussion

A series of novel derivatives were designed using simple and effective strategies including amino acid substitution and N-terminal truncation. The 3D model of the parent acidocin J1132β peptide revealed two helixes connected with an unstructured turn with an imperfect amphipathic structure on a helical wheel projection. Generally, an idealized facial or perfect amphipathic structure comprising of uninterrupted cationic and hydrophobic portion promotes higher membrane-penetrating activity with improved selectivity^36–38^. Therefore, an imperfect and perfect amphiphilic α-helix peptide derivatives of acidocin J1132β were designed by substitution with positively charged (lysine and arginine) and hydrophobic (tryptophan and valine) amino acids, and truncation of unstructured regions. The step-by-step replacement with similar types of amino acid residue in targeted positions (basic amino acid in polar faces and hydrophobic amino acid in nonpolar faces) gradually increased the mean hydrophobic moment (0.065–0.637), an indicator of α-helical peptide amphipathicity^39^. A9 and A11 peptides with perfect uninterrupted hydrophobic and cationic faces provided enhanced activity and selectivity towards a vast array of Gram-positive and -negative bacteria. It is thought that improved amphipathicity through balancing of peptide hydrophobicity and hydrophilicity plays a vital role in antimicrobial activity and selectivity^38^. Significantly, A11 (with tryptophan’s substitution on the polar/nonpolar interface) showed the strongest antibacterial activity with no obvious hemolytic activity. Tryptophan appears to have a significant role in antimicrobial activity by anchoring AMPs to the membrane of bacteria via their aromatic side chain and affecting the interface region of lipid bilayer and disturbing the internal structure of cellular membranes^40–42^. Our study supports previous reports that amphipathicity with side-chained amino acids on hydrophobic/hydrophilic faces, hydrophobicity and net charge serve as key factors in designing membrane-disruptive AMPs^43,44^.

The most potent acidocin J1132β derivative, A11, displayed high antibacterial activity against *S*. Typhimurium including drug-resistant and its monophasic variant strains. To further investigate its promise as a food preservative, safety of the peptide was examined. The peptide's ability to disrupt human red blood cells was assessed. A11 caused less than 5% hemolysis, even at a concentration of 250 μg/ml. This result, along with no cytotoxic effect toward L929 mouse fibroblast cells, was supportive of its safety. Positively charged AMPs interact with negatively charged phospholipid headgroups [such as phosphatidylglycerol (PG), cardiolipin (CL) or phosphatidylserine (PS)] of bacterial membranes through electrostatic interactions resulting in their displacement or structural modification^45^. In contrast, the outer membrane of mammalian cells is embellished in zwitterionic neutral phospholipids (such as phosphatidylcholine, phosphatidylethanolamine and sphingomyelin) and other neutral components such as cholesterol. This is the reason why AMPs specifically target membranes of microbes and not mammalian cells^17,46^. Although melittin exhibited potent antimicrobial activity, it caused extensive hemolytic activity toward human red blood cells and dramatically reduced mouse fibroblast cell survival even at low concentration, similar to recently published report^47^. Higher hydrophobicity was associated with excessive hemolytic activity. It is very crucial to optimize the hydrophobicity and amphipathicity of AMPs to obtain potency and high selectivity^31^.

By CD analysis, A11 was a random coil in aqueous or hydrophobic (50% TFE) environments and converted to an amphiphilic α-helix structure when exposed to negatively charged mimetic environments (30 mM SDS). It had a lower tendency to form an α-helix structure in a hydrophobic environment than in a negatively charged environment. This result implied that the alteration influences the segregation of charged amino acid residues within the hydrophobic parts leading to an amphipathic structure recognized as having a crucial role in the AMPs interaction with bacterial membranes^48^. The anionic region of the bacterial membrane plays a key role in inducing the conformational change of AMPs which is essential for the electrostatic interaction between the negatively charged membranes and cationic AMPs^49^. Adopting a secondary structure in negatively charged environments may increase the antimicrobial activity of A11.

As previously mentioned, the initial step of bacterial killing by AMPs is their interaction with and attachment to bacterial outer membranes through electrostatic interactions^50^. Most AMPs kill bacterial cells by disrupting membranes via the formation of pores or ion channels^50^. After reaching a certain concentration, AMPs insert into the hydrophobic core of bacterial membranes leading to the disruption^4^. It was noticed that the permeabilization or pore formation is transient. Melittin, a bee venom AMP, causes transient pore formation in the cell membranes of *E. coli*. This membrane breach re-seals over a 21-min interval. Eventually, melittin induces re-permeabilization of cell membranes, causing leakage of intracellular contents and cell death^51^. The antibacterial mechanisms of A11 toward *S.* Typhimurium ATCC 13311 were determined and compared with melittin. *S*. Typhimurium ATCC 13311 (NCTC 74), the type strain of *S.* Typhimurium, is quinolone-susceptible and often used as a reference strain in multidrug resistance studies^52^ and for antibacterial activity determinations of new compounds^53,54^. A11 bound, penetrated and damaged the membranes of *S*. Typhimurium via permeabilization and depolarization during the initial incubation period.

Confirming the results of flow cytometry, TEM revealed that A11 killing was associated with membrane permeabilization and depolarization by pore-formation at an early stage (4 h). However, a non-lytic mode of killing was apparent after prolonged incubation (12 h). Although many AMPs act on bacterial membranes and induce cell lysis^55^, non-membranolytic action appears superior since this AMP capacity is less likely to induce bacterial resistance^56^. Additionally, lytic AMPs are more toxic due to outer membrane destabilization and disruption of the Gram-negative bacteria, and subsequent release of endotoxins or lipopolysaccharides (LPS) causing toxic damage to human tissues^57^. In this study, A11 killed the bacterial cells by both lytic and non-lytic modes, likely depending on time and concentration of peptide inside cells. Non-lytic AMPs can translocate into the cytoplasm and affect the function of important cellular components such as nucleic acids resulting in cell death^58^. Peptide F1, a peptide from *Tibetan kefir*, exhibits antibacterial activity against *E. coli* via multiple targets, both cell membranes and genomic DNA*.* DNA aggregated by F1 appears to interfere with essential intracellular functions and lead to bacterial death^59^. Buforin II killed bacterial cells via DNA binding after translocation into cells without significant membrane damage^60^. Interactions of the A11 peptide with the genomic DNA of *S*. Typhimurium were analyzed and showed that it bound DNA forming a peptide–DNA complex as demonstrated by slow or no migration through agarose gel (compared to free genomic DNA). Our DNA-binding study revealed that A11 had DNA-binding ability, suggesting a non-lytic killing activity by interfering with, or affecting, nucleic acids. However, intracellular targeting should be further assessed to confirm killing and determine its mechanism.

Use of a peptide as a food preservative requires stability under many environmental conditions, including a wide range of pH, temperatures, and various salt concentrations. In general, pH has an important effect on the antibacterial activity of AMPs by affecting the initial charge-charge interactions. Net charge of the peptide or of the bacterial target may change and directly affect binding^61^. A11 was much less effective against *S*. Typhimurium at low pH (pH 3). Previous research showed that an excessive positive charge can negatively affect the antibacterial activity of positively charged AMPs^4^. The antibacterial activity of A11 was retained even after heating at 100 °C for 1 h. Therefore, it is considered a heat-stable peptide, the same as its parent peptide (acidocin J1132β)^5^. Several studies show that most of bacteriocins produced by *L. acidophilus* are heat-stable (such as lactacin F from *L. acidophilus* 11088, acidocin 8912 from *L. acidophilus* TK8912, acidocin B from *L. acidophilus* M46, acidocin J1229 from *L. acidophilus* JCM 1229, and acidocin J1132 from *L. acidophilus* JCM 1132)^5,62–64^. A11 was stable at low salt concentrations but inactive at higher concentrations. This is consistent with reports that the antibacterial activity of AMPs is typically reduced at higher salt concentrations^61^. This inhibitory effect of salts may be due to the positively charged molecules of salt interrupting the electrostatic interaction and binding between peptide and bacterial membrane^25,65^. But this salt effect is likely not an obstacle since salts themselves inhibit microbial growth and are already used as food preservatives^66^.

Combination of antimicrobial agents for food preservation is a common approach to the inhibition of foodborne pathogens, and reduces the dosage of the individual agents^12^. The interaction between A11 and nisin against ten selected drug-resistant *S. enterica* isolates was found to be synergistic. The ability of A11 to enhance the efficacy of nisin to control drug-resistant *S. enterica* strains suggests use of the combination as a broad-spectrum food preservative. Our data provide evidence that the combination of nisin with A11 is a potentially effective approach for controlling foodborne bacterial growth and expanding the shelf-life of food products.

## Conclusion

A novel antimicrobial peptide, A11, was modified from acidocin J1132β by truncation and amino acid substitution. It exhibited potent inhibitory activity against *S*. Typhimurium, including drug-resistant strains, and its monophasic variants, with very low toxicity. It eliminated *S*. Typhimurium via membrane attachment and penetration causing depolarization and inducing transient permeabilization, and/or intracellular mechanisms. A11 displayed thermal stability at temperatures up to 100 °C. Significantly, it was synergistic with nisin against *S*. Typhimurium in vitro. The efficacy of this combination in vivo or in food modeling should be included in future studies to advance product development for the food industry.

## Supplementary Information

Supplementary Information.

## Data Availability

All data generated or analyzed during this study are included in this published article and its supplementary information files.

## References

[1] Majowicz SE (2010). International collaboration on enteric disease “Burden of Illness” studies. The global burden of nontyphoidal *Salmonella gastroenteritis*. Clin. Infect. Dis..

[2] Haeusler GM, Curtis N (2013). Non-typhoidal *Salmonella* in children: Microbiology, epidemiology and treatment. Adv. Exp. Med. Biol..

[3] Rai M, Pandit R, Gaikwad S, Kövics G (2016). Antimicrobial peptides as natural bio-preservative to enhance the shelf-life of food. J. Food Sci. Technol..

[4] Wang S, Zeng X, Yang Q, Qiao S (2016). Antimicrobial peptides as potential alternatives to antibiotics in food animal industry. Int. J. Mol. Sci..

[5] Garcia-Gutierrez E, Mayer MJ, Cotter PD, Narbad A (2019). Gut microbiota as a source of novel antimicrobials. Gut Microbes.

[6] Gálvez A, López RL, Pulido RP, Burgos MJG (2014). Application of lactic acid bacteria and their bacteriocins for food biopreservation. Food Biopreserv..

[7] Parada JL, Caron CR, Adriane BP, Socol CR (2007). Bacteriocins from lactic acid bacteria: Purification, properties and use as bio preservatives. Braz. Arch. Biol. Technol..

[8] Kitagawa N, Otani T, Inai T (2019). Nisin, a food preservative produced by *Lactococcus lactis*, affects the localization pattern of intermediate filament protein in HaCaT cells. Anat. Sci. Int..

[9] Administration FAD (1988). Nisin preparation; Affirmation of GRAS status as a direct human food ingredient. Fed. Reg..

[10] Delves-Broughton J (2008). Use of the natural food preservatives, nisin and natamycin, to reduce detrimental thermal impact on product quality. In Pack Processed Foods.

[11] Davidson PM, Zivanovic S (2003). The use of natural antimicrobials. Food Preserv. Tech..

[12] Gautam N, Sharma N (2009). Bacteriocin: Safest approach to preserve food products. Indian J. Microbiol..

[13] Roshanak S, Shahidi F, Yazdi FT, Javadmanesh A, Movaffagh J (2020). Evaluation of antimicrobial activity of buforin I and nisin and the synergistic effect of their combination as a novel antimicrobial preservative. J. Food Prot..

[14] Churklam W, Chaturongakul S, Ngamwongsatit B, Aunpad R (2020). The mechanisms of action of carvacrol and its synergism with nisin against *Listeria monocytogenes* on sliced bologna sausage. Food Control.

[15] Shwaiki LN, Lynch KM, Arendt EK (2021). Future of antimicrobial peptides derived from plants in food application—a focus on synthetic peptides. Trends Food Sci. Tech..

[16] Klubthawee N, Adisakwattana P, Hanpithakpong W, Somsri S, Aunpad R (2020). A novel, rationally designed, hybrid antimicrobial peptide, inspired by cathelicidin and aurein, exhibits membrane-active mechanisms against *Pseudomonas aeruginosa*. Sci. Rep..

[17] Qu P (2016). The central hinge link truncation of the antimicrobial peptide fowlicidin-3 enhances its cell selectivity without antibacterial activity loss. Antimicrob. Agents Chemother..

[18] Zhang SK (2016). Design of an alpha-helical antimicrobial peptide with improved cell-selective and potent anti-biofilm activity. Sci. Rep..

[19] Deraz SF, Karlsson EN, Khalil AA, Mattiasson B (2007). Mode of action of acidocin D20079, a bacteriocin produced by the potential probiotic strain, *Lactobacillus acidophilus* DSM 20079. J. Ind. Microbiol. Biotechnol..

[20] Modiri S (2020). Multifunctional acidocin 4356 combats *Pseudomonas aeruginosa* through membrane perturbation and virulence attenuation: Experimental results confirm molecular dynamics simulation. Appl. Environ. Microbiol..

[21] Huoy L, Pornruangwong S, Pulsrikarn C, Chaturongakul S (2014). Molecular characterization of Thai *Salmonella enterica* serotype Typhimurium and serotype 4,5,12:i:- reveals distinct genetic deletion patterns. Foodborne Pathog. Dis..

[22] Clinical and Laboratory Standards Institute (CLSI). Performance Standards for Antimicrobial Susceptibility Testing, 28th ed. *CLSI supplement M100*. (2018).

[23] Nizet V (2017). The accidental orthodoxy of Drs. Mueller and Hinton. EBioMedicine.

[24] Andrews JM (2001). Determination of minimum inhibitory concentrations. J. Antimicrob. Chemother..

[25] Ma Z (2016). Insights into the antimicrobial activity and cytotoxicity of engineered alpha-helical peptide amphiphiles. J. Med. Chem..

[26] Rabanal F (2015). A bioinspired peptide scaffold with high antibiotic activity and low in vivo toxicity. Sci. Rep..

[27] Shao C (2018). Central beta-turn increases the cell selectivity of imperfectly amphipathic alpha-helical peptides. Acta Biomater..

[28] Jia B (2020). High cell selectivity and bactericidal mechanism of symmetric peptides centered on D-Pro-Gly pairs. Int. J. Mol. Sci..

[29] Bag A, Chattopadhyay RR (2017). Synergistic antibacterial and antibiofilm efficacy of nisin in combination with p-coumaric acid against food-borne bacteria *Bacillus cereus* and *Salmonella* typhimurium. Lett. Appl. Microbiol..

[30] Zhao L, Au JL, Wientjes MG (2010). Comparison of methods for evaluating drug-drug interaction. Front. Biosci. (Elite Ed).

[31] Chen Y (2007). Role of peptide hydrophobicity in the mechanism of action of alpha-helical antimicrobial peptides. Antimicrob. Agents Chemother..

[32] Falcigno L (2021). Key physicochemical determinants in the antimicrobial peptide RiLK1 promote amphipathic structures. Int. J. Mol. Sci..

[33] Won HS, Jung SJ, Kim HE, Seo MD, Lee BJ (2004). Systematic peptide engineering and structural characterization to search for the shortest antimicrobial peptide analogue of gaegurin 5. J. Biol. Chem..

[34] Polanco C (2012). Characterization of selective antibacterial peptides by polarity index. Int. J. Pept..

[35] da Cunha NB (2017). The next generation of antimicrobial peptides (AMPs) as molecular therapeutic tools for the treatment of diseases with social and economic impacts. Drug Discov..

[36] Jacob B, Park IS, Bang JK, Shin SY (2013). Short KR-12 analogs designed from human cathelicidin LL-37 possessing both antimicrobial and antiendotoxic activities without mammalian cell toxicity. J. Pept. Sci..

[37] Khara JS (2017). Disruption of drug-resistant biofilms using de novo designed short α-helical antimicrobial peptides with idealized facial amphiphilicity. Acta Biomater..

[38] Wiradharma N, Sng MY, Khan M, Ong ZY, Yang YY (2013). Rationally designed α-helical broad-spectrum antimicrobial peptides with idealized facial amphiphilicity. Macromol. Rapid Commun..

[39] Eisenberg D, Weiss RM, Terwilliger TC (1982). The helical hydrophobic moment: A measure of the amphiphilicity of a helix. Nature.

[40] Bi X, Wang C, Ma L, Sun Y, Shang D (2013). Investigation of the role of tryptophan residues in cationic antimicrobial peptides to determine the mechanism of antimicrobial action. J. Appl. Microbiol..

[41] Chan DI, Prenner EJ, Vogel HJ (2006). Tryptophan- and arginine-rich antimicrobial peptides: Structures and mechanisms of action. Biochim. Biophys. Acta.

[42] Feng X (2020). The critical role of tryptophan in the antimicrobial activity and cell toxicity of the duck antimicrobial peptide DCATH. Front Microbiol..

[43] Klubthawee N, Aunpad R (2020). A thermostable, modified cathelicidin-derived peptide with enhanced membrane-active activity against *Salmonella enterica* serovar Typhimurium. Front Microbiol..

[44] Yin LM, Edwards MA, Li J, Yip CM, Deber CM (2012). Roles of hydrophobicity and charge distribution of cationic antimicrobial peptides in peptide-membrane interactions. J. Biol. Chem..

[45] Giuliani A, Pirri G, Nicoletto S (2007). Antimicrobial peptides: An overview of a promising class of therapeutics. Open Life Sci..

[46] Silva JP, Appelberg R, Gama FM (2016). Antimicrobial peptides as novel anti-tuberculosis therapeutics. Biotechnol. Adv..

[47] Askari P, Namaei MH, Ghazvini K, Hosseini M (2021). *In vitro* and *in vivo* toxicity and antibacterial efficacy of melittin against clinical extensively drug-resistant bacteria. BMC Pharmacol. Toxicol..

[48] Liang Y, Zhang X, Yuan Y, Bao Y, Xiong M (2020). Role and modulation of the secondary structure of antimicrobial peptides to improve selectivity. Biomater. Sci..

[49] Brogden KA (2005). Antimicrobial peptides: Pore formers or metabolic inhibitors in bacteria?. Nat. Rev. Microbiol..

[50] Kumar P, Kizhakkedathu JN, Straus SK (2018). Antimicrobial peptides: Diversity, mechanism of action and strategies to improve the activity and biocompatibility in vivo. Biomolecules.

[51] Yang Z, Choi H, Weisshaar JC (2018). Melittin-induced permeabilization, re-sealing, and re-permeabilization of *E. coli* membranes. Biophys. J..

[52] Terabayashi Y (2014). First complete genome sequence of *Salmonella enterica* subsp. enterica serovar Typhimurium strain ATCC 13311 (NCTC 74), a reference strain of multidrug resistance, as achieved by use of PacBio single-molecule real-time technology. Genome Announc..

[53] Silva F, Ferreira S, Queiroz JA, Domingues FC (2011). Coriander (*Coriandrum sativum* L.) essential oil: Its antibacterial activity and mode of action evaluated by flow cytometry. J. Med. Microbiol..

[54] Singh AP, Prabha V, Rishi P (2013). Value addition in the efficacy of conventional antibiotics by nisin against *Salmonella*. PLoS One.

[55] Seyfi R (2020). Antimicrobial peptides (AMPs): Roles, functions and mechanism of action. Int. J. Pept. Res. Ther..

[56] Cardoso MH (2019). Non-lytic antibacterial peptides that translocate through bacterial membranes to act on intracellular targets. Int. J. Mol. Sci..

[57] Hernández-Cortez C (2017). Food poisoning caused by bacteria (food toxins). Poisoning Specific Toxic Agents Novel Rapid Simplified Tech Anal..

[58] Li YM, Xiang Q, Zhang QH, Huang YD, Su ZJ (2012). Overview on the recent study of antimicrobial peptides: Origins, functions, relative mechanisms and application. Peptides.

[59] Miao J (2016). Inhibitory effects of a novel antimicrobial peptide from kefir against *Escherichia coli*. Food Control.

[60] Park CB, Yi KS, Matsuzaki K, Kim MS, Kim SC (2000). Structure-activity analysis of buforin II, a histone H2A-derived antimicrobial peptide: The proline hinge is responsible for the cell-penetrating ability of buforin II. PNAS.

[61] Walkenhorst WF, Klein JW, Vo P, Wimley WC (2013). pH dependence of microbe sterilization by cationic antimicrobial peptides. Antimicrob. Agents Chemother..

[62] Muriana PM, Klaenhammer TR (1991). Purification and partial characterization of lactacin F, a bacteriocin produced by *Lactobacillus acidophilus* 11088. Appl. Environ. Microbiol..

[63] Tahara T (1992). Purification and some properties of acidocin 8912, a novel bacteriocin produced by *Lactobacillus acidophilus* TK8912. Biosci. Biotechnol. Biochem..

[64] Ten Brink B, Minekus M, van der Vossen JM, Leer RJ, Huis in’t Veld JHJ (1994). Antimicrobial activity of lactobacilli: Preliminary characterization and optimization of production of acidocin B, a novel bacteriocin produced by *Lactobacillus acidophilus* M46. J. Appl. Bacteriol..

[65] Kandasamy SK, Larson RG (2006). Effect of salt on the interactions of antimicrobial peptides with zwitterionic lipid bilayers. Biochim. Biophys. Acta Biomembr..

[66] Henney JE (2010). Preservation and Physical Property Roles of Sodium in Foods. Strategies to Reduce Sodium Intake in the United States.

