# Supplementary material for: A novel designed membrane-active peptide for the control of foodborne Salmonella enterica serovar Typhimurium
Source: Sci Rep. 2023 Mar 2;13:3507. doi: 10.1038/s41598-023-30427-z (PMC9981719; doi:10.1038/s41598-023-30427-z)
Supplement: Supplementary file 1 — Supplementary Information. [file 41598_2023_30427_MOESM1_ESM.docx]

**Supplementary Data**

**
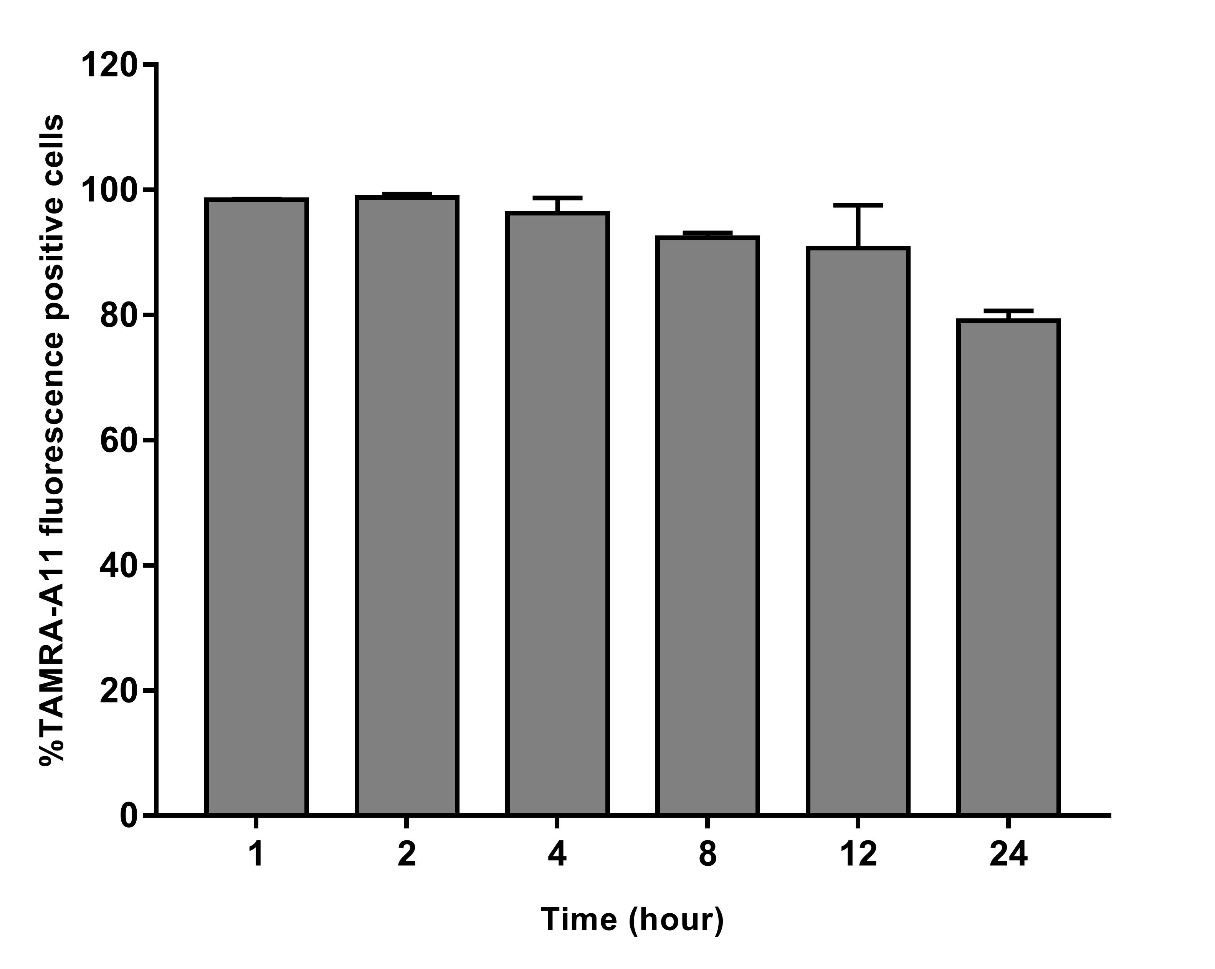
**

**Supplementary Figure 1.** The membrane-binding activity of TAMRA-labeled A11 at 1 x MIC to *S. enterica* serovar Typhimurium ATCC 13311 after 1, 2, 4, 8, 12 and 24 h of incubation as determined by flow cytometry. The experiments were done in triplicate and the data were represented as the mean ± SD.

**Supplementary Figure 2.** Flow cytometry analysis of *S. enterica* serovar Typhimurium ATCC13311 after treatment with A11 peptide at 1 x MIC. Untreated *S. enterica* serovar Typhimurium without PI and BOX (A) and untreated *S. enterica* serovar Typhimurium with PI and BOX stained as a negative control (B). The effect of thermal lysis at 70°C for 30 min as a positive control (C) and A11 at 1 x MIC for 1, 2, 4, 8, 12 and 24 h (D–I) on membrane permeability (PI) and membrane potential (BOX). The percentage of cell populations that fell in each gate are shown in the four corners of each plot.
